# Cyclosporine A binding to COX-2 reveals a novel signaling pathway that activates the IRE1α unfolded protein response sensor

**DOI:** 10.1038/s41598-018-34891-w

**Published:** 2018-11-12

**Authors:** Jody Groenendyk, Tautvydas Paskevicius, Hery Urra, Clement Viricel, Kui Wang, Khaled Barakat, Claudio Hetz, Lukasz Kurgan, Luis B. Agellon, Marek Michalak

**Affiliations:** 1 Department of Biochemistry, University of Alberta, Edmonton, Alberta T6G 2S7 Canada; 2 Biomedical Neuroscience Institute, Faculty of Medicine, University of Chile, Santiago, Chile; 3 Center for Geroscience, Brain Health and Metabolism (GERO), University of Chile, Santiago, Chile; 4 Program of Cellular and Molecular Biology, Institute of Biomedical Sciences, University of Chile, Santiago, Chile; 5 Faculty of Pharmacy and Pharmaceutical Sciences, University of Alberta, Edmonton, Alberta T6G 2S7 Canada; 6 School of Mathematical Sciences and LPMC, Nankai University, Tianjin, People’s Republic of China; 7 Department of Immunology and Infectious Diseases, Harvard School of Public Health, Boston, MA 02115 USA; 8 The Buck Institute for Research in Aging, Novato, CA 94945 USA; 9 Department of Computer Science, Virginia Commonwealth University, Richmond, 23284 USA; 10 School of Human Nutrition, McGill University, Ste. Anne de Bellevue, Quebec, H9X 3V9 Canada

## Abstract

Cyclosporine, a widely used immunosuppressant in organ transplantation and in treatment of various autoimmune diseases, activates the unfolded protein response (UPR), an ER stress coping response. In this study we discovered a new and unanticipated cyclosporine-dependent signaling pathway, with cyclosporine triggering direct activation of the UPR. COX-2 binds to and activates IRE1α, leading to IRE1α splicing of XBP1 mRNA. Molecular interaction and modeling analyses identified a novel interaction site for cyclosporine with COX-2 which caused enhancement of COX-2 enzymatic activity required for activation of the IRE1α branch of the UPR. Cyclosporine-dependent activation of COX-2 and IRE1α in mice indicated that cyclosporine-COX-2-IRE1α signaling pathway was functional *in vivo*. These findings identify COX-2 as a new IRE1α binding partner and regulator of the IRE1α branch of the UPR pathway, and establishes the mechanism underlying cytotoxicity associated with chronic cyclosporine exposure.

## Introduction

ER stress is caused by many intrinsic or extrinsic factors that disturb ER homeostasis and functions, leading to activation of the unfolded protein response (UPR), an ER stress coping response. ER stress has been implicated in the occurrence of diverse diseases including cancer, neurodegeneration, and inflammation to name a few. Recent observations also suggest that chronic ER stress plays a pathogenic role during renal fibrosis, impacting kidney biology^1^. The UPR involves distinct components designed to re-establish the protein synthetic machinery, including translational attenuation, transcriptional activation of genes encoding chaperones and components of the ER-associated degradation (ERAD), and activation of apoptotic and autophagy pathways^2–4^. Inositol-requiring enzyme 1α (IRE1α) is an ER transmembrane protein kinase and the most evolutionary conserved ER stress sensor and component of the UPR. This protein has endoribonuclease activity that splices the mRNA encoding the transcription factor XBP1, resulting in a form of mRNA that directs the translation of XBP1s, the stable form of the transcription factor. XBP1s induces the expression of genes involved in many aspects of the secretory pathway, including protein folding, ERAD, and protein quality control^5^. In addition, IRE1α degrades selected mRNAs and microRNAs through a process referred as regulated IRE1-dependent decay (RIDD), contributing to cell death, inflammation and other biological processes. Sustained activation of IRE1α signaling leads to apoptosis and autophagy possibly through uncontrolled RIDD, JNK activation, miRNA deregulation and other complementary mechanisms^6–8^.

Cyclosporine is a small polypeptide from the fungus *Tolypocladium inflatum* and has been widely used as an immunosuppressant in organ transplantation and in treatment of various autoimmune diseases^9^. Primarily, cyclosporine inhibits the immune response upon inflammatory stimuli by binding to cyclophilin A, a cytoplasmic peptidyl prolyl isomerase enzyme, with the complex associating with and inhibiting calcineurin, a protein serine/threonine phosphatase. This interaction prevents the de-phosphorylation of NF-AT, its translocation to the nucleus and the stimulation of genes responsible for the activation of T-cells^10^. Cyclosporine also blocks the c-Jun N-terminal kinase (JNK) and p38 pathways triggered by antigen recognition^11^ and has been identified to bind to endoplasmic reticulum (ER) localized cyclophilin B^12,13^, and the mitochondrial localized cyclophilin D^14^. Long term treatment with cyclosporine induces a variety of side effects including hyperlipidemia, hyperuricemia, gingival hyperplasia, but arterial hypertension, organ fibrosis and chronic nephrotoxicity are the most serious complications^15,16^.

Cyclooxygenases (COX) are members of a heme enzyme family that catalyze a cyclooxygenase and a peroxidase reaction to produce prostaglandins^17^. COX-1 (human gene symbol *PTGS1*) is ubiquitously and constitutively expressed in mammalian tissues and cells, whereas COX-2 (human gene symbol *PTGS2*) is inducible and is present in mammalian tissues at variable levels. It is thought that inflammatory stimuli induce expression of COX-2 at the site of inflammation, increasing the abundance of prostaglandins and proteases. COX-2 is localized at membranes of the ER and the nuclear envelope^18–21^. Increased COX-2 activity is associated with renal tissue damage and poor outcome for kidney transplant patients^22–24^. The COX-2 enzyme is upregulated during cardiac allograft rejection^25^ and inhibition of COX-2 improves transplanted cardiac function and outcome^26^.

Enhanced COX-2 expression and production of prostaglandins has been recently associated with induction of ER stress^27,28^ but the molecular mechanism(s) responsible for the COX-dependent activation of ER stress is not known. In this study, we discovered that COX-2 is a new target for cyclosporine and that COX-2 is a novel component of the UPR. Cyclosporine enhances COX-2 enzymatic activity required for the activation of IRE1α. These findings revealed a new and unanticipated cyclosporine-dependent signaling pathway and provide a mechanism for how cyclosporine and COX-2 activate the IRE1α branch of the UPR pathway.

## Results

### COX-2 mediates cyclosporine-dependent effects on the ER stress coping response

**Figure 1:**
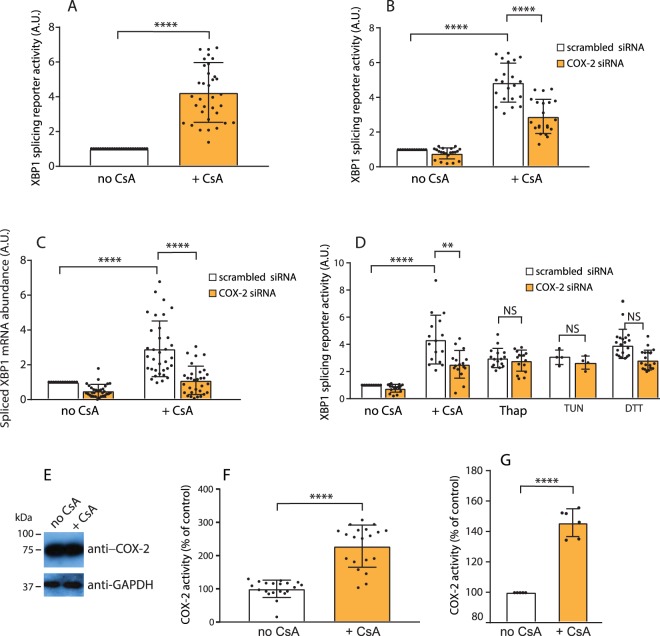
Silencing of COX-2 affects IRE1α activity. (**A**) HEK293 cells were transfected with the IRE1α splicing reporter plasmid and treated for 24 hours with 20 µM cyclosporine A (+*CsA*). ****p-*value* < 0.0001 (n = 35). (**B**) Cells were transfected with the IRE1α splicing reporter plasmid in combination with siRNA for COX-2 (*COX-2 siRNA*) or control scrambled siRNA (*scrambled siRNA*) followed by treatment for 24 hours with 20 µM cyclosporine A (+*CsA*). ****p-*value* < 0.0001 (n = 21). (**C**) Q-PCR quantitative analysis of spliced endogenous XBP1 in HEK293 cells transfected with siRNA for COX-2 (*COX-2 siRNA*) or control scrambled siRNA (*scrambled siRNA*) followed by treatment with 20 µM cyclosporine A (+*CsA*). ****p-*value* < 0.0001 (n = 34). (**D**) HEK293 cells were transfected with siRNA for COX-2 (*COX-2 siRNA*) or control scrambled siRNA (*scrambled siRNA*) and with the XBP1 splicing reporter vector. Cells were treated for 24 hours with 20 µM cyclosporine A (+*CsA*), thapsigargin (0.5 µM) (*Thap*), tunicamycin (5 µg/ml) (*Tun*) or DTT (1 mM). ****p-value < 0.0001, **p-*value* = 0.0014 (n = 16); NS, not significant. **(E)** Immunoblot analysis of HEK293 cells treated for 24 hours with 20 µM cyclosporine A (+*CsA*). Blots were probed with anti-COX-2 and anti-GAPDH antibodies. (**F**) HEK293 cells were incubated with 20 µM cyclosporine A (+*CsA*) followed by analysis of COX-2 peroxidase activity. ****p-value < 0.0001 (n = 20). (**G**) Peroxidase activity of purified COX-2 protein was monitored in the absence (*no CsA*) and presence of 20 µM cyclosporine A (+*CsA*). ****p-value < 0.0001 (n = 6). The images of (**E**) shown are cropped. The full-length blots are shown in Suppl. Fig. S10.

Cyclosporine activates UPR^29,30^ as shown by the cyclosporine-dependent activation of IRE1α-XBP1 pathway (Fig. 1A) and the activation was dose responsive (Suppl. Fig. S1A). A major unanswered question remains with respect to the identity of the molecular factors responsible for cyclosporine-dependent effects on UPR. To address this, we carried out a genome-wide siRNA screen for genes required for the activation or inactivation of IRE1α, a component of the UPR and ER stress coping response^31^. We monitored the activity of IRE1α using the XBP1 mRNA splicing reporter based on the expression of luciferase coupled to direct splicing of the mRNA for XBP1^31,32^. Filtered analysis of 400 genes identified 6 candidates whose silencing produced inactivation of IRE1α reporter activity in response to cyclosporine (Table 1). Unexpectedly, one of these genes (*PTGS2*) encodes an ER-associated inducible prostaglandin endoperoxidase synthase 2, (prostaglandin G/H synthase and cyclooxygenase) commonly referred to as COX-2^17^. Next, we carried out validation experiments using an unbiased siRNA approach^31^. Quantitative (Q)-PCR analysis of human kidney HEK293 cells treated with cyclosporine revealed that cyclosporine did not interfere with siRNA-dependent silencing of COX-2 (Suppl. Fig. S1B). The siRNA was also effective in attenuating COX-2 protein abundance (Suppl. Fig. S1C). To validate the specificity of our siRNA analysis, a pool of siRNA as well as individual siRNAs directed against COX-2 were used and showed that all siRNAs reduced COX-2 mRNA abundance (Suppl. Fig. S1D). We then tested for effects of silencing of COX-2 mRNA on IRE1α-dependent splicing of XBP1 mRNA, a measure of IRE1α activity and an indicator of UPR activation^31^. Silencing COX-2 reduced IRE1α reporter activity in response to cyclosporine (Fig. 1B), suggesting that cyclosporine exerted its effects on IRE1α reporter activity in HEK293 kidney cells *via* a COX-2-dependent pathway. Knocking down COX-2 also reduced the splicing of endogenous XBP1 mRNA (Fig. 1C). Importantly, cyclosporine treatment in combination with siRNA for COX-2 did not compromise the ability of cyclosporine to inhibit calcineurin phosphatase activity (Suppl. Fig. S2A). Moreover, the combined treatment had no effect on the abundance of TNF-α, JNK1 and IFN-γ mRNAs, markers of inflammation (Suppl. Fig. S2B–D). Silencing of COX-2 did not have any effect on the abundance of IRE1α mRNA or protein as well as on the abundance of total XBP1 mRNA (Suppl. Fig. S3).

**Table 1:** Candidate genes identified in the siRNA screen of NIH3T3 cells treated with cyclosporine.

Ref Sequence	Gene Symbol	Gene ID	Full Gene Name	Z score
NM_009984	13039	Ctsl	cathepsin L	−1.26
NM_011159	19090	Prkdc	protein kinase, DNA activated, catalytic polypeptide	−1.14
NM_172053	271127	Adamts16	disintegrin-like and metalloprotease (reprolysin type) with thrombospondin type 1 motif, 16	−1.03
NM_009809	12365	Casp14	caspase 14	−0.9
NM_013913	30924	Angptl3	angiopoietin-like 3	−0.73
NM_011198	19225	Ptgs2	prostaglandin-endoperoxide synthase 2 (COX-2)	−2.35

To determine whether downstream gene targets of the XBP1s were altered, we measured the mRNA abundance of EDEM1, ERdj4 and mTOR genes in cells treated with cyclosporine in the absence or presence of COX-2 silencing. The mRNAs of EDEM1 and ERdj4 genes showed reduced abundance upon silencing of COX-2 in the presence of cyclosporine (Suppl. Fig. S4A,B). In addition, mTOR gene, which is upregulated upon ER stress^33^, also showed down regulation of mRNA abundance in the presence of cyclosporine and silencing of COX-2 (Suppl. Fig. S4C). We also examined whether silencing of COX-2 affected cyclosporine-dependent activation of the UPRE reporter system that is dependent on XBP1s transcriptional activity^34^. There was significant reduction in UPRE reporter activity in cells treated with cyclosporine during COX-2 silencing (Suppl. Fig. S4D).

Next, we tested whether the effects of COX-2 on IRE1α-dependent XBP1 splicing were associated with accumulation of misfolded proteins using a classical inducers of protein misfolding and the UPR, thapsigargin, tunicamycin and DTT^4^. In sharp contrast to the cyclosporine treatment (Fig. 1D, +*CsA*), stimulation of ER stress with thapsigargin, tunicamycin or DTT did not have any effect on COX-2-dependent stimulation of XBP1 mRNA splicing (IRE1α activity) (Fig. 1D). We concluded that cyclosporine mediated its effects on IRE1α activity *via* COX-2.

Immunoblot analysis revealed no significant changes in the abundance of COX-2 protein in HEK293 cells treated with cyclosporine (Fig. 1E), however, endogenous COX-2 peroxidase activity was increased in the presence of cyclosporine (Fig. 1F). Purified COX-2 protein activity was also increased in the presence of cyclosporine (Fig. 1G).

Next, we carried out XBP1 splicing reporter assays in the presence of inhibitors of COX-2 cyclooxygenase or peroxidase activity. Cyclosporine binding to COX-2 was not affected by celecoxib or sodium ortho-vanadate (Suppl. Fig. S5A). Celecoxib, which inhibits COX-2 cyclooxygenase activity^35^, had no effect on the XBP1 splicing activity in the absence or presence of COX-2 silencing (Suppl. Fig. S5B). However, addition of sodium ortho-vanadate, an inhibitor of COX-2 peroxidase activity, resulted in significant reduction of XBP1 splicing in the presence of COX-2, but not when COX-2 was silenced (Suppl. Fig. S5B).

### Cyclosporine binds COX-2

**Figure 2:**
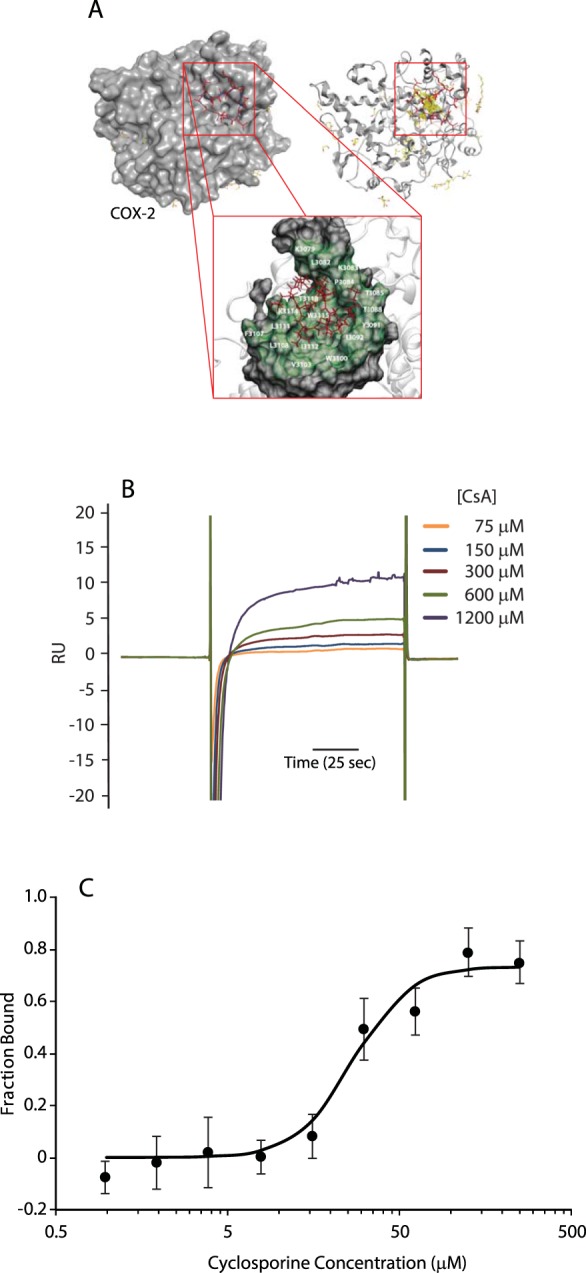
Cyclosporine A interacts with COX-2. (**A**) Putative binding mode of cyclosporine *A* in the COX-2 structure (PDB ID: 1DDX chain D). The structure COX-2 is shown in gray in surface representation in the top left corner and in cartoon representation in the top right corner. Cyclosporine A is represented using red (carbon atoms) and blue (nitrogen atoms) sticks, while the other ligands are shown in yellow (carbon atoms), blue (nitrogen atoms) and red (hydrogen atoms). The cartoon structure reveals position of all ligands while the surface representation shows ligands on the same side of COX-2 where cyclosporine A binds. The red box indicates position of the putative binding site of cyclosporine A on the surface of COX-2. An enlarged view of the binding site where the interacting surface is rendered in green and the residues that are predicted to interact with cyclosporine A are given in white font is shown at the bottom. The numbering system for the residues was based on chain D in the 1DDX structure of COX-2 (additional 3 in front of AA number). (**B**) COX-2 was immobilized on a CM5 chip followed by flow of increasing concentrations of cyclosporine A *(CsA)* and analyzed by SPR; K_D_ = 895 ± 224 nM. SPR analysis was carried out in triplicate. (**C**) Analysis of cyclosporine A interaction with COX-2 using Label Free Microscale Thermophoresis (MST). MST analysis was carried out in triplicate.

How can cyclosporine exert its effects on IRE1α via COX-2? We hypothesized that cyclosporine directly interacts with COX-2. To test this hypothesis, we first carried out molecular modelling and docking of COX-2 with cyclosporine using Autodock 4^36^. Previous research from our laboratories has determined that cyclosporine may interact with proteins other than cyclophilins using a combination of docking and prediction techniques^13^. Using this analysis, we identified a possible cyclosporine binding site on COX-2 with a favorable binding energy. We obtained molecular details of binding between COX-2 and cyclosporine utilizing an integrative computational pipeline that combines methods for ligand binding prediction, molecular dynamics simulations and molecular docking. This identified a putative binding site localized to the surface along with the identity of binding amino acid residues (Fig. 2A, bottom panel). The model predicts the cyclosporine peptide occupying a deep groove on the COX-2 surface, which was formed by amino acid residues located in a segment of COX-2 between Pro^84^ and Thr^118^ (Fig. 2A). The interaction was characterized by a favorable putative docking energy at −36.3 K_cal_/mol, estimated based on molecular docking. Strikingly, the site of the interaction did not overlap with any of the 72 distinct sites of interactions of COX-2 with its 36 ligands identified to date. It is therefore unlikely that binding of cyclosporine to COX-2 would affect interactions with the other known ligands.

To define the occurrence of direct interactions between cyclosporine and COX-2, we applied surface plasmon resonance (SPR) and microscale thermophoresis (MST) techniques. For cyclosporine binding, we used cyclophilin A and carbonic anhydrase as positive and negative controls, respectively (Suppl. Fig. S6 for MST and Suppl. S7A for SPR). In agreement with the modeling analysis (Fig. 2A), cyclosporine bound to COX-2 in a dose dependent manner (K_D_ = 895 ± 224 nM) (Fig. 2B). Direct binding of cyclosporine to COX-2 was confirmed using MST (Fig. 2C). Importantly, cyclosporine binding to COX-2 did not induce unfolding of COX-2 as revealed by tryptophan fluorescence and thermal stability analysis of COX-2 protein and cyclosporine bound COX-2 (Suppl. Fig. S7B,C). Taken together, these data show that cyclosporine interacted with COX-2, resulting in increased COX-2 peroxidase activity.

### COX-2 interacts with IRE1α to stimulate XBP1 mRNA splicing

**Figure 3:**
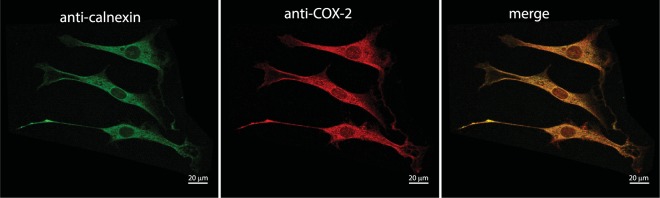
Immunolocalization of COX-2. Immunostaining of NIH3T3 cells with antibodies against calnexin, an ER marker, and against COX-2. Pearson’s coefficient for the merge image = 0.75 ± 0.02 (n = 3).

**Figure 4:**
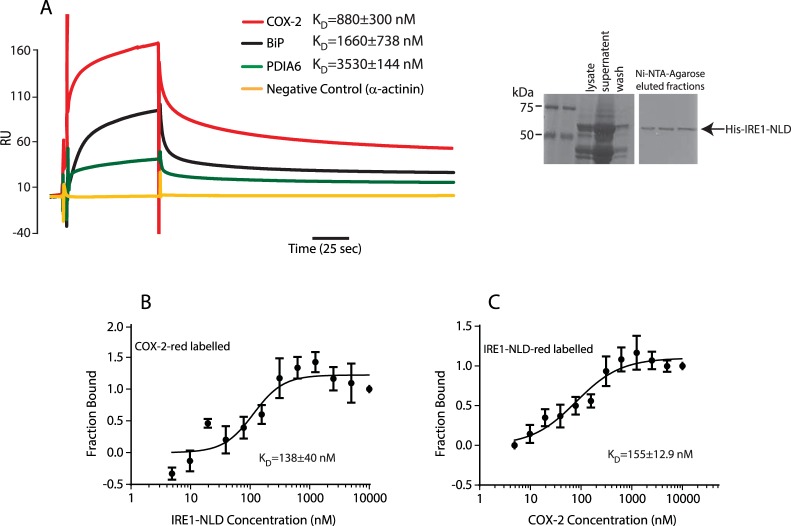
COX-2 interacts with IRE1α. (**A**) Ten µM COX-2, BiP and PDIA6 were flowed over immobilized IRE1-NLD. K_D_ = 880 ± 300 nM for COX-2 binding to IRE1-NLD is indicated in the Figure. A negative control (α-actinin) showed no binding to IRE1-NLD. SPR analysis was carried out in triplicate. *Right panel*: SDS-PAGE of Ni-NTA-Agarose chromatography purified IRE1-NLD. Fractions eluted with 300 mM imidazole are indicated. Protein samples were separated on the same SDS-PAGE and stained with Coomassie blue, the middle lanes were removed for the sake of clarity. (**B**) Purified COX-2 protein was covalently labeled with a red fluorescent tag and incubated with increasing amounts of purified IRE1-NLD protein followed by Microscale Thermophoresis (MST). (**C**) The reverse was performed with purified IRE1-NLD protein covalently labeled with a red fluorescent tag and incubated with increasing amounts of purified COX-2 followed by Microscale Thermophoresis (MST). MST analysis was carried out in triplicate. The image of (**A**) shown is cropped. The full-length gels are shown in Suppl. Fig. S11.

**Figure 5:**
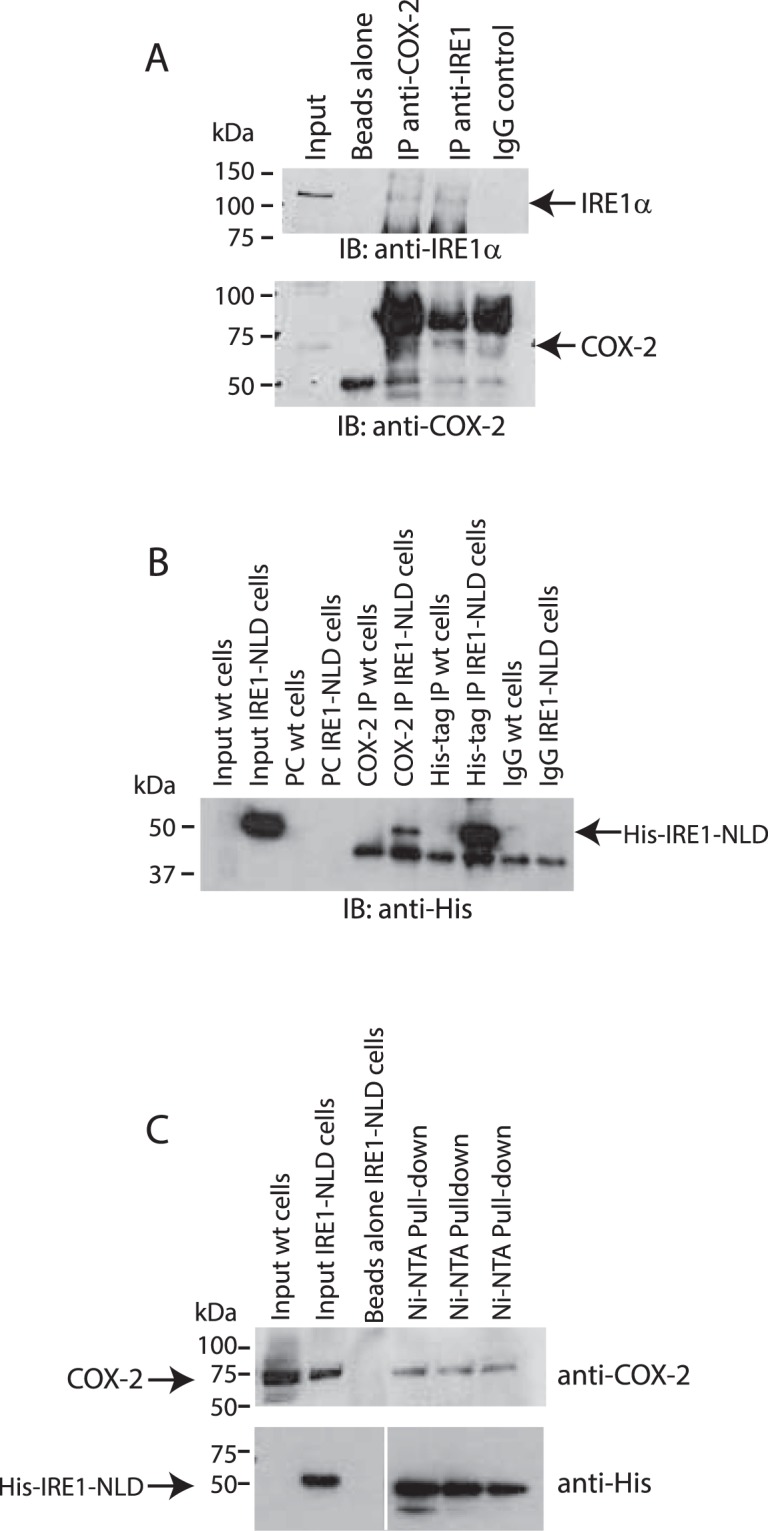
COX-2 interacts with IRE1α *in vivo*. (**A**) Immunoprecipitation (*IP*) of endogenous IRE1α from HEK293 cells with anti-IRE1α or anti-COX-2 -antibodies. Immunoblot (*IB*) analysis was carried out with anti-IRE1α or anti-COX-2 antibodies. The location of IRE1α and COX-2 are indicated by the arrows. Immunoprecipitation experiments were performed in triplicate with representative blots shown. (**B**) His-tagged ER luminal domain of IRE1α (*IRE1-NLD*) was expressed in COS-1 cells followed by immunoprecipitation with anti-COX-2, anti-His-tag antibodies or IgG. Immunoblot (*IB*) analysis was carried out with anti-His antibodies. The location of IRE1-NLD is indicated by the arrow. Immunoprecipitation experiments were performed in triplicate with representative blot shown. (**C**) Pull-down of COX-2 in COS-1 cells expressing His-tagged IRE1-NLD. Upper blot was probed with anti-COX-2 antibodies. The lower blot was probed with anti-His-tag antibodies. Protein samples were separated on the same SDS-PAGE, the middle empty lanes were removed from the lower blot for the sake of clarity. Pull-down assay was performed in triplicate. The images of (**A**–**C**) shown are cropped. The full-length gels/blots are shown in Suppl. Figs S12 and S13.

The activity of IRE1α has been shown to be regulated by several proteins that associate with the ER luminal or cytoplasmic domains^37^. Thus, we asked whether COX-2, an ER associated protein^18–21^ (Fig. 3) affected XBP1 splicing via a direct interaction with IRE1α. Indeed, SPR (Fig. 4A) and MST (Fig. 4B,C) analyses showed that COX-2 bound tightly to the ER luminal domain of IRE1α (IRE1-NLD) *in vitro* with a K_D_ of 880 nM. COX-2 interaction with IRE1-NLD was not sensitive to the presence of cyclosporine (Suppl. Fig. S8A) and cyclosporine did not bind to IRE1-NLD (Suppl. Fig. S8B). Next, we performed immunoprecipitation and pull-down assays to detect the interaction between COX-2 and IRE1α in living cells. COX-2 co-immunoprecipitated with endogenous IRE1α (Fig. 5A, upper panel) and conversely, IRE1α antibody immunoprecipitated endogenous COX-2 (Fig. 5A, lower panel). His tagged IRE1-NLD was also immunoprecipitated with an anti-COX-2 antibodies (Fig. 5B). We then carried out pull-down experiments with the His-tagged luminal domain of IRE1α. His-tagged IRE1α ER luminal domain (IRE1-NLD) was expressed in COS-1 cells followed by Ni-NTA column/His-tag pull-down and immunoblot analysis with anti-His and anti-COX-2 antibodies (Fig. 5C). In three independent experiments, His-tagged IRE1-NLD pulled down COX-2 from COS-1 cell extracts (Fig. 5C), indicating that the luminal domain of IRE1α and COX-2 formed complexes in cells. We concluded that cyclosporine binds to COX-2, and in turn COX-2 interacts with IRE1α.

### COX-2 peroxidase activity is necessary for the modulation of IRE1α activity

**Figure 6:**
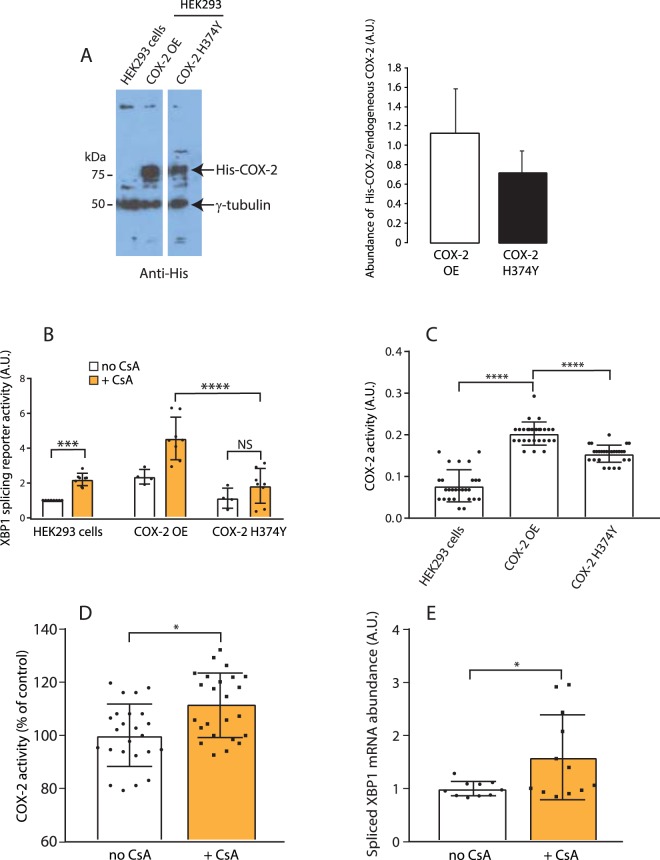
COX-2 peroxidase activity was needed to regulate the IRE1α arm of UPR. (**A**) Immunoblot analysis of HEK293 cells and HEK293 cells transfected with His-COX-2 or His-COX-2 H374Y expression vector with anti-His and anti-γ-tubulin antibodies. Protein samples were separated on the same SDS-PAGE and the middle lane was removed for the sake of clarity. Quantitative analysis of the abundance of COX-2 and COX-2 mutant proteins is indicated in the graph (n = 4). (**B**) HEK293 (*HEK293*) cells, and stably transfected COX-2 overexpressing HEK293 cells (*COX-2 OE*) or COX-2 H374Y mutant (*COX-2 H374Y*) were transfected with the IRE1α splicing reporter followed by treatment for 24 hours with 20 µM cyclosporine A (+*CsA*). ***p-value < 0.0006 (n = 8), ****p-value < 0.0001. (n = 8). (**C**) HEK293 cells, and HEK293 cells overexpressing COX-2 (*COX-2 OE*) or COX-2 H374Y mutant (*COX-2 H374Y*) were analyzed for COX-2 peroxidase activity. *p-value = 0.0309, ***p-value < 0.0003 (n = 20). (**D**) COX-2 peroxidase activity in kidneys from cyclosporine treated mice. *p-value = 0.0069 (n = 6). (**E**) Q-PCR quantitative analysis of spliced endogenous XBP1 in kidneys harvested from cyclosporine treated mice. *p-*value* < 0.0012 (n = 6). The images of (**A**) shown are cropped. The full-length gels/blots are shown in Suppl. Fig. S14.

The increase in COX-2 peroxidase activity after binding cyclosporine (Fig. 1F,G) suggested that COX-2 peroxidase activity may play a role in IRE1α activation. Thus, we generated a cell line overexpressing His-tagged COX-2 (HEK293 COX-2 OE) as well as a cell line overexpressing the His-tagged COX-2 H374Y mutant (Fig. 6A), which has a 300-fold reduction in peroxidase activity^38^.

Addition of cyclosporine to HEK293 cells increased XBP1 mRNA splicing as expected (Fig. 6B). Overexpression of wild-type COX-2 increased the XBP1 splicing activity reporter of IRE1α indicating that COX-2 has a basal level peroxidase activity, which can be further amplified by addition of cyclosporine (Fig. 6B). COX-2 peroxidase activity was also significantly increased in cells overexpressing COX-2 (Fig. 6C). In contrast, cells overexpressing the COX-2 H374Y mutant showed no further increase in the XBP1 splicing activity reporter in cells treated with cyclosporine (Fig. 6B) and showed reduced peroxidase activity when compared to COX-2 overexpressing cells (Fig. 6C). MST analysis indicated that both wild-type COX-2 and COX-2 H374Y bound to IRE1-NLD (Suppl. Fig. S9) indicating that the H374Y mutation did not interfere with the ability of COX-2 to bind to the IRE1α luminal domain. Taken together, these findings indicated that cyclosporine binds to and activates COX-2 and that the peroxidase activity of COX-2 is required for activation of IRE1α.

To establish the effects of cyclosporine on COX-2 activity and XBP1 splicing *in vivo*, mice were fed cyclosporine followed by analysis of COX-2 activity and XBP1 splicing. In agreement with the *in vitro* analyses (Fig. 2B,C) both COX-2 activity (Fig. 6D) and XBP1 splicing (Fig. 6E), a measure of IRE1α activity, were increased in the kidneys in response to cyclosporine treatment (Fig. 6D,E). This indicated that cyclosporine induced COX-2 activity and in turn resulting in the activation of UPR signaling *in vivo*.

## Discussion

In this study we discovered a new and unanticipated cyclosporine-dependent signaling pathway that leads to the activation of the UPR, an ER stress coping response. First, we identified COX-2 as a novel target for cyclosporine and then established that COX-2 interacts with IRE1α, an ER associated stress sensor and component of the UPR, to activate IRE1α. Activation of IRE1α by COX-2 was independent of the accumulation of misfolded proteins since ER Ca^2+^ depletion (thapsigargin treatment), which causes the accumulation of misfolded proteins, did not affect COX-2-dependent stimulation of IRE1α, nor did cyclosporine induce mis-folding of COX-2. However, we found that the binding of cyclosporine to COX-2 enhanced its peroxidase activity which in turn is necessary for the activation of IRE1α. Importantly, COX-2 and IRE1α activities were also increased *in vivo* in mice treated with cyclosporine indicating that cyclosporine-COX-2-IRE1α signaling pathway was also functional *in vivo*. These findings provide an explanation for how cyclosporine activates UPR and establishes COX-2 as a new IRE1α binding partner.

Understanding the molecular events controlling IRE1α activation is crucial to assess the connection between the ER stress coping response and cell/organism physiology and pathology^4^. IRE1α signaling is controlled by its interaction with different proteins (including phosphatases, kinases, apoptosis-related proteins and the cytoskeleton) that modulate its activity through binding to its cytoplasmic domain^2–4^. In contrast, regulation of IRE1α activity by components of the ER luminal environment remains poorly understood. BiP, an ER resident chaperone, associates with IRE1α, resulting in inactivation of the pathway, whereas under stress conditions, BiP dissociates from IRE1α to promote its dimerization, increased endonuclease activity and activation of the UPR^2–4,39^. PDIA6, an oxidoreductase and ER luminal resident protein, has been identified as a binding partner with IRE1α^31,40^ that together with changes in ER luminal Ca^2+^ and expression of miR-322, modulates IRE1α activity^31^. Recently, another ER resident protein, Hsp47 was identified as regulator of IRE1α^41^. In the present study, we report COX-2 as a new partner and modulator of the IRE1α branch of the UPR. COX-2 is an inducible cyclooxygenase ER-associated protein^18–21^ that has two enzymatic activities, namely cyclooxygenase activity that converts arachidonic acid to prostaglandin G2 and peroxidase activity that reduces prostaglandin G2 to prostaglandin H2^17^. COX-2 is thought to play a role in the pathophysiology of a variety of disorders including renal disease^23^, central nervous system diseases^42^, allograft rejection^25^, cancer^43^ and other inflammatory diseases^20^, all of which involve the UPR^4^. We found that enzymatically active COX-2 is essential for COX-2 mediated cyclosporine induction of the IRE1α branch of the UPR. Interestingly, it has been shown that COX-2 induces oligomerization of β-amyloid protein in Alzheimer’s disease and this also requires COX-2 peroxidase activity^44,45^. We propose that cyclosporine-dependent induction of COX-2 peroxidase activity represents a novel, cyclosporine-induced point of control of IRE1α signaling.

Dysregulated cellular stress coping responses, including UPR and genome damage response, are drivers of multiple pathological conditions, ranging from cancer, neurodegeneration, inflammatory, and metabolic disorders^4,46–48^. In the case of ER stress, IRE1α initiates the most conserved signaling branch of the UPR which affects many cellular processes including cellular energetics^49,50^, inflammation^51^, immunity^52^, angiogenesis^53^, aging and longevity^54^, and neurodegeneration^4,31,55–58^. However, the molecular mechanism(s) responsible for cyclosporine-dependent activation of the UPR have long remained poorly understood. It has been shown that cyclosporine functions as an immunosuppressor by binding to cyclophilin A and the resulting complex associates with and inhibits calcineurin thereby preventing calcineurin-dependent de-phosphorylation of NF-AT which is needed for nuclear translocation^10^. Complications associated with prolonged use of cyclosporine in transplant patients include hyperlipidemia, hypertension and severe nephrotoxicity^59^. It is well documented that the UPR is activated in solid organ transplantation^60,61^. The results of the present study uncovered molecular mechanisms of how cyclosporine activates UPR by demonstrating the ability of cyclosporine to regulate the RNA splicing activity of IRE1α via COX-2. This finding provides, in part, an explanation for the observed toxicity resulting from prolonged cyclosporine exposure.

## Materials and Methods

### Ethics

All methods were carried out in accordance with relevant guidelines and regulations and approved by the Biosafety Officers at the Department of Environment, Health and Safety at the University of Alberta. All animal experiments were carried out according to the University of Alberta Animal Policy and Welfare Committee and the Canadian Council on Animal Care Guidelines (Permit AUP297). All animal experimentation was carried out working closely with University of Alberta animal facility staff and veterinarian.

### Chemicals

The siRNA library was from Ambion and Dharmafect Duo was from GElifesciences. Cyclosporine, thapsigargin, tunicamycin, DTT, celecoxib and other chemicals were from Sigma. Cyclophilin A and carbonic anhydrase were purchased from OriGene.

### siRNA library screen

A genome wide druggable library screen (400 genes) was carried out in NIH-3T3 mouse fibroblast cells as described previously^31^. Briefly, NIH-3T3 cells were transfected with siRNA (20 nM) for each druggable gene and pRL-IXFL plasmid (0.1 μg) using Dharmafect Duo (Qiagen) for 48 h, followed by treatment with 20 µM cyclosporine for 24 h. Cells were harvested, monitored for luciferase activity and results were analyzed using Z-score. The top 50 hits (increased luciferase activity) and bottom 50 hits (reduced luciferase activity) for each treatment were robot picked and a second validation screen was carried out. Results were analyzed for significance and several genes were selected for validation^31^. From the cyclosporine treated positive hits (Table 1), ptgs2 (COX-2) was selected for further validation.

### Cell Culture

HEK293 cells (human female embryonic kidney cells; https://web.expasy.org/cellosaurus/CVCL_0045) were maintained under standard tissue culture conditions, including 5% CO_2_ with high humidity. Tissue culture media included 10% fetal bovine serum in DMEM (Sigma). Cell transfection, analysis of cell viability and luciferase assays were carried out as described previously^31^.

### Plasmids and silencing

The pRL-IXFL XBP1 splicing reporter was a generous gift from Dr. R. Kaufman (Sanford Burnham Medical Discovery Institute) and contains an internal *Renilla* control and the nucleotide sequence encoding XBP1 followed by firefly luciferase separated by an internal ribosomal entry site (IRES) initiation region^32^. This reporter will only generate firefly luciferase if the XBP1 sequence is spliced, bringing the firefly luciferase sequence to the correct reading frame.

Silencing was carried out as described in^31^.

PTGS2 (COX-2) siRNA (Qiagen Cat # GS5743)

5′-AACACCGGAAUUUUUGACAAG-3′;

5′-UUGGAACGUUGUGAAUAACAU-3′;

5′-UAGGGUAGAAUCACCUGUAAA-3′;

5′-ACGCUUUAUGCUGAAGCCCUA-3′.

Scrambled control siRNA (Qiagen, Cat #: 1022076). The COX-2 mammalian expression plasmid was purchased from Origene and was cloned in the pCMV6 plasmid. The single site mutation H374Y was generated by Genscript and was cloned in the pCMV6 plasmid for mammalian expression. HEK293 cells were transfected with COX-2 OE or COX-2 H374Y expression vectors to generate stable cell lines. Protein expression was monitored using Immunoblot analysis.

The Cignal Luciferase Reporter Assay UPRE was from Qiagen and is composed of a ratio 40:1 with a *Renilla* control plasmid. Analysis was carried out according to manufacturer’s protocol.

### XBP1 splicing and mRNA analyses

The quantitative analysis of spliced XBP1 transcripts in HEK293 cells was utilized to identify XBP1 specific splicing^62^. The forward primer sequence; 5′-CCGCAGCAGGTGCAGG-3′ (human), reverse primer sequence; 5′-GAGTCAATACCGCCAGAATCCA-3′ (human). The forward primer spans the XBP1 splice site, therefore only annealing when the 26 bases are removed, and is combined with the reverse primer sequence to quantitate the amount of XBP1 splicing that is occurring. Q-PCR analysis was comprised of a reaction (20 μl) which contained 500 nM forward and reverse primers, 100 ng cDNA templates made from total RNA, and 1× SYBR Green Supermix (Quanta). Thermal cycling parameters were 95 °C for 10 min; 95 °C for 20 s, 58 °C 15 s, and 72 °C for 15 s and repeated for 40 cycles. A threshold was set at the logarithmic linear phase when it could be distinguished from the background (crossing point). The threshold was expressed as a cycle number (Ct) and was normalized to glyceraldehyde 3-phosphate dehydrogenase (GAPDH). The quantitation was performed using the ΔΔCt method. mRNA analysis using Q-PCR was carried out as described previously^31^.

### Reverse transcriptase-PCR (RT-PCR) and Quantitative-PCR (Q-PCR)

To monitor the levels of mRNA, cells were harvested at day three after siRNA transfection using the RNeasy kit (Qiagen) according to manufacturer’s protocol and concentration was measured spectrophotometrically. Total RNA (200 ng) was subsequently used in RT-PCR to generate cDNA for each sample. For monitoring of the mRNA levels, the cDNA was diluted 5-fold, with 2 µl of cDNA used in subsequent PCR reactions with primers targeting controls, selected genes, and ER stress markers and was performed in duplicate on at least three separate occasions. Quantitative PCR was performed using a RotorGeneQ (Qiagen) and a RotorGene 3000 rapid thermal cycler system (GE Life Sciences) according to the manufacturer’s instructions. The following nucleotide primers were used for Q-PCR analyses:

h-PTGS2:

5′-ATATGTTCTCCTGCCTACTGGAA-3′;

5′-GCCCTTCACGTTATTGCAGATG-3′

h-GAPDH:

5′-AGGGCTGCTTTTAACTCTGGT-3′;

5′-CCCCACTTGATTTTGGAGGGA-3′

h-IFN-γ:

5′-CCAACGCAAAGCAATACATGA-3′;

5′-CCTTTTTCGCTTCCCTGTTTTA-3′

h-TNF-α:

5′-GGAGAAGGGTGACCGACTCA-3′;

5′-CTGCCCAGACTCGGCAA-3′

h-JNK1:

5′-GCGCGGATCCTTGCTTGCCATCATGAGCAG-3′;

5′-GCGCGGATCCCAGACGACGATGATGATGGA-3′

h-mTOR:

5′-CTGGGACTCAAATGTGTGCAGTTC-3′

5′-GAACAATAGGGTGAATGATCCGGG-3′

h-EDEM1:

5′-GAATGGCTGAGGAGGAGATTAC-3′

5′-CTACACGTGGGAATAGGAAGATG-3′

h-ERdj4:

5′-TCTGGAGGTATAGAGGGCATATAA-3′

5′-TGTGAGAGAAGGATGGTAAGAATG-3′

### Computational analysis

Computation of the putative binding pose of cyclosporine on the surface of COX-2. The computation includes four steps: (1) initial assessment of the propensity for binding between cyclosporine and COX-2; (2) MD simulations to equilibrate structures and sample conformational spaces of cyclosporine and COX-2; (3) docking between equilibrated conformations of cyclosporine and COX-2 to establish binding pose and estimate docking energy; and (4) mapping of other ligands into the same conformation of COX-2.

### Assessment of propensity of binding between COX-2 and cyclosporine

We estimate propensity for COX-2-cyclosporine binding and the approximate position of the binding site using a recent ligand binding predictor ILbind^63^. This method predicts propensity for binding for a given small ligand and protein structure based on the knowledge of structures of the known ligand–target protein complexes. Our analysis extends from our recent study that investigated binding of cyclosporine with a large set of over 9000 human and mouse proteins and that has shown that ILbind accurately finds protein targets of this compound^13^. We computed the propensities for binding cyclosporine to each of the 93 conformations of COX-2 from the Protein Data Bank (PDB). All but one COX-2 structure secured scores of 0.55 and above, while 50 of the 93 have scores over 0.6. These are relatively high scores given that the scores for proteins that are known to bind cyclosporine are in the range of 0.6 and above^13^. We selected the two highest scoring conformations of COX-2, 1DDX chain D and 4COX chain A, for which the propensities equal 0.625 and 0.622, respectively, for the subsequent analysis. We limited our analysis to the two structures due to a relatively high computational cost of running molecular dynamics and docking. We also used ILbind to provide the estimated position of the site of the COX-2-cyclosporine interaction which we utilized to constrain the search space for docking.

### Molecular dynamics simulations

Both the ligand and the binding interface in the interacting protein may experience conformational changes resulting from flexibility of their structures. We considered these changes by docking an ensemble of protein structures to another ensemble of ligand structures. The two ensembles were generated using the molecular dynamics simulations. For cyclosporine we used ten distinct conformations that we developed in the earlier study^13^. For COX-2 we performed two molecular dynamics simulations, one for each selected structure, to relax and equilibrate their structures and to create the conformational ensemble. We used a protocol that was recently applied in similar studies^64–67^. Briefly, we calculated the protonation states of all ionizable residues using PDB2PQR^68^ followed by adding the proper concentrations of sodium and chloride ions to neutralize the systems. Each solvated system was then minimized, heated with heavy restraints on the backbone atoms, equilibrated for 100 ps with a gradual removal of the restraints and finally run for production 6 ns molecular dynamics simulations. We carried out the M molecular dynamics D simulations using the NAMD program^69^, with the all-hydrogen AMBER99SB force field^70^ simulated in a 12Å-wide buffer of water molecules, at a mean temperature of 300 K and physiological pH (pH 7). The root mean square deviation (RMSD) values sampled over the time of the simulations revealed that the 4COX structure experienced more atomic fluctuations (RMSD fluctuated around 2 Å) compared to 1DDX (RMSD fluctuated around 1.4 Å), indicating that the COX-2 conformation in 1DDX was more rigid. Both structures were fully equilibrated after 3 ns of molecular dynamics simulations. The last 3 ns were used to construct conformational ensemble of COX-2 structures for the subsequent docking. We extracted 20 representative conformations of COX-2, 10 from each 1DDX and 4COX.

### Docking simulations

We validated the binding of cyclosporine to COX-2 in the vicinity of the binding site identified by ILbind and predicted its binding mode within this site using docking. We performed two hundred independent docking simulations that consider all combinations of the 10 conformations of cyclosporine and 20 conformations of COX-2. We employed a recently developed relaxed complex scheme methodology to account for both the target and ligand flexibility during docking simulations^71^ and use ZDOCK for docking^72–74^. We ranked each of the COX-2-cyclosporine docked complexes using scores generated by ZDOCK and retained the top ten hits for 1DDX and top ten hits for 4COX for further analysis. We refined the docked structures for the twenty best hits using MD simulations and used the more accurate molecular mechanics-Poisson Boltzmann surface area (MM-PBSA) method^75^, compared to the scores generated by ZDOCK, to estimate the binding energies of the resultant protein-ligand complexes. We adopted the MD simulations protocol described above and used it to generate an ensemble by storing the trajectories every 10 ps for the docking energy calculations. We utilized the same parameters as described in the literature^13,65,67^ for the MM-PBSA calculations. Briefly, the total free energy *G* was estimated as the sum of the average molecular mechanical gas-phase energies *E*_*MM*_, solvation free energies *G*_*solv*_, and entropy contributions *-TS*_*solute*_ of the binding reaction: *G* = *E*_*MM*_ + *G*_*solv*_ − *TS*_*solute*_. The molecular mechanical energy of each snapshot was calculated using the SANDER module of AMBER10. The solvation free energy was estimated as the sum of electrostatic solvation free energy, calculated by the finite-difference solution of the Poisson–Boltzmann equation in the Adaptive Poisson-Boltzmann Solver and non-polar solvation free energy, calculated from the solvent-accessible surface area algorithm. The solute entropy was approximated using the normal mode analysis. Due to high computational costs to compute the entropy contribution, we used 100 snapshots for this purpose. The best docking energy among the ten considered hits for the 1DDX structure of COX-2 was −36.3 Kcal/mol, which is substantially lower by about 15 kcal/mol than the best docking energy for 4COX (−21.1 Kcal/mol). To compare, the docking energies for proteins that are known to bind cyclosporine are in similar range at −42.1 Kcal/mol for FAB fragment IGG1-kappa, −55.1 for Cyclophilin A, and −60.4 for Cyclophilin C^13^. These results suggest that cyclosporine likely binds COX-2 with a higher preference toward the 1DDX COX-2 conformation.

### Mapping of ligands into the COX-2 structure

773 interactions were collected between 36 distinct ligands and COX-2 from the 92 structures of COX-2 that are deposited in the PDB. These interactions were superimposed onto the 1DDX structure of COX-2. Since some ligands appear in multiple structures of COX-2 and they occupy the same or very similar position relative to COX-2, they were grouped together and represented by a single instance. The grouping was accomplished using hierarchical clustering based on distances between all instances of the same ligand. 72 clusters were obtained which correspond to 72 unique interactions between the 36 ligands and COX-2.

### SPR analysis

SPR technology was employed to monitor the interaction of cyclosporine with purified COX-2 protein (BIACore T200, GE Life Sciences). To study the interaction, purified COX-2, cyclophilin A (positive control) and carbonic anhydrase (negative control) proteins were separately immobilized to carboxymethylated dextran on a gold surface with cyclosporine flowing over the immobilized ligand surface at varying concentrations. Briefly, a CM5 chip was activated using a 1:1 dilution of EDC:NHS as previously described^31^. Purified protein was diluted in 10 mM NaAc, pH 5 and injected over the activated CM5 chip followed by the blocking solution of 1 M ethanolamine, pH 9. A reference lane with no ligand coupled was generated to subtract background binding. The running buffer was composed of 10 mM Hepes, pH 7.2, 150 mM NaCl, 1 mM EDTA, 0.005% P20, and 2% ethanol. The cyclosporine was diluted in 100% ethanol and the dilution series was carried out with experiments performed in triplicate. For each experiment, the signal was corrected against the control surface response to eliminate any refractive index changes due to buffer change. The data were collected at 25 °C at a flow rate of 30 µl/min to minimize mass transfer effects. Positive (cyclophilin) and negative controls (carbonic anhydrase) were included. Kinetic analysis was performed using the BiaEvaluation software (GE Life Sciences) with a 1:1 Langmuir binding model. Association and dissociation rates and affinity (K_D_) were calculated for each experiment and averaged. The binding response signals in RUs were continuously recorded and presented graphically as a function of time. For SPR analysis of COX-2 interaction with IRE1-NLD, the carboxymethylated dextran (CMD) surface of a CM5 chip was activated using a 1:1 dilution of EDC:NHS as previously described^31^. Purified IRE1-NLD was captured at a flow rate of 5 μl/min to a total of ~1500 Response Units (RU). Uncoupled amine reactive sites on the CMD surface were then blocked by an injection of ethanolamine. The CM5 chip was normalized and prepared for kinetic analysis. COX-2 in a buffer containing 10 mM Hepes, pH 7.4, 150 mM NaCl, and 0.005% P20 at concentrations from 0 to 10000 nM and was passed over the sensor surface at a flow rate of 30 μl/min. Kinetic analysis was performed in triplicate. All experiments and analysis were conducted on a BIACore T200 instrument (GE Life Sciences).

### MicroScale Thermophoresis (MST)

MST was carried out using a Monolith NT.115 instrument (NanoTemper). To evaluate COX-2 binding to IRE1-NLD, an increasing concentration of purified COX-2 protein (0–10 µM) was incubated with RED-labeled purified IRE1-NLD protein (NanoTemper; as per manufacturer’s protocol). The reverse was also performed, with RED-labeled purified COX-2 incubated with an increasing concentration of purified IRE1-NLD. Positive (cyclophilin A) and negative controls (carbonic anhydrase) were included. To evaluate cyclosporine binding to COX-2, a Monolith Label Free instrument (NanoTemper) was used with an increasing concentration of cyclosporine (0–500 µM) incubated with purified COX-2 protein. Positive (cyclophilin A) and negative controls (carbonic anhydrase) were included. Experiments were carried out in a buffer containing 10 mM Hepes, pH 7.4, 125 mM NaCl, and 0.005% Tween-20, with 2% ethanol included in cyclosporine experiments. Experiments were done in triplicate and data evaluation was performed using the Monolith software (NanoTemper).

### Immunoblot and immunostaining analyses

HEK-293 cell lysates were collected and analyzed using SDS-PAGE and Immunoblot analysis. Antibodies specific for COX-2 (Abcam, ab52237), GAPDH (ThermoFisher Sci MA5-15738), ATF4 (Abcam ab23760), γ-Tubulin (ThermoFisher Sci MA1-850), IRE1α (Abcam, ab37073), and His-Tag (ThermoFisher Sci MA1-21315) were used.

Tryptophan fluorescence. Fluorescence intensity of 1 µM COX-2 protein in the absence or presence of 20 µM cyclosporine was carried out in 10 mM Mops, pH 7.5, 150 mM KCl and 3 mM MgCl_2_ at 25 °C using the Photon Technology International PTI TC-125 fluorometer. The sample was excited at 286 nm and an emission scan was monitored from 295–450 nm. For thermal stability analysis, 1 µM COX-2 protein was incubated in the absence or presence of 20 µM cyclosporine and the temperature was ramped from 25 °C to 60 °C with excitation at 286 nm and emission at 340 nm using the Photon Technology International PTI TC-125 fluorometer. Experiments were done in triplicate.

For immnostaining NIH3T3 were fixed in ice-cold methanol for 5 min, washed 3 times for 5 min with PBS and permeabilized with 100 µM digitonin for 10 min. Cells were incubated with 10% normal goat serum (NOVEX life technologies; catalog number PCN5000) diluted in PBS for 1 h followed by 60 min incubation with primary antibodies: rabbit anti-calnexin (Enzo; catalog # ADi-SPA-860-F) at dilution 1:200 or mouse anti COX-2 (BD Biosciences; catalog #r 610203) at dilution 1:50. Cells were washed and incubated with goat ant-mouse (Alexa Fluor 594; life technologies; catalog # A21135) at dilution 1:50 or goat ant-rabbit (Alexa Fluor 488; life technologies; catalog # A11034) at dilution 1:50. Slides were visualized using a Leica TCS SP5 microscope.

### Calcineurin activity assay

HEK293 cells were transfected with siRNA for the negative control or siRNA for COX-2 and were resuspended in calcineurin activity assay buffer. The calcineurin activity assay was performed as previously described^76^. Insoluble material was pelleted, and a protein assay was performed on the supernatant. Assay Buffer (50 mM Tris, pH 6.5, 2 mM EDTA) was prepared fresh by the addition of 5 mM DTT final concentration and 16.7 mM pNPP (*p*-nitrophenyl phosphate). Twenty µl of samples were aliquoted in 96 well plates in triplicate followed by addition of 180 µl of fresh Assay buffer and incubated at room temperature until colour changed to yellow. The plate was read at 405 nm using a spectrophotometric microplate reader (Molecular Devices) and normalized to protein concentration. Experiments were done in triplicate.

### Enzymatic Assays

To monitor COX-2 enzymatic activity, a COX-2 activity assay was performed (Cayman Chemicals) according to manufacturer’s protocols. The COX-2 activity assay monitors the peroxidase activity of COX-2 by assaying the appearance of oxidized N,N,N′,N′-tetramethyl-*p*-phenylenediamine (TMPD) at 590 nm. It was used with purified enzyme as well as with HEK293 cellular lysates preincubated with varying concentrations of cyclosporine. The COX-2 activity assay was performed in triplicate.

### Purification of IRE1-NLD, COX-2 protein and COX-2 H374Y mutant protein

Cells were transfected with the pED-IRE1-NLD-His6-KDEL expression vector and purified by Ni-NTA-Agarose^77^. Briefly, COS-1 cells were transfected with IRE1-NLD expression vector, harvested and lysed in a buffer containing 25 mM Tris-Cl, pH 8.0, 150 mM NaCl, 0.5% Nonidet P-40, and 0.5% sodium deoxycholate. Cell lysates were centrifuged at 20,000 × g for 15 min, and cell extracts were used for protein purification. Ni-NTA-Agarose chromatography was carried out using a buffer containing 50 mM Tris-Cl, pH 8.0, 300 mM NaCl, and 10 mM imidazole. The IRE1-NLD protein was eluted with 300 mM imidazole and concentrated. COX-2 cDNA was purchased from OriGene and cloned into pET-22b containing a 6His-tag for expression in LEMO21 *E. coli* cells. Single site mutagenesis to generate the COX-2 H374Y mutant was performed by GenScript and transformed *E. coli* were grown to an OD of 0.3–0.6 and induced with 0.1 mM IPTG. Cells were harvested, crushed and the supernatant passed over a Ni-NTA-Agarose column and eluted with 300 mM imidazole. Fractions were combined, concentrated and buffer exchanged for further purification using a Resource-Q column. Purified COX-2 protein and COX-2 H374Y mutant protein were eluted using a high salt gradient, with fractions combined and concentrated.

### IRE1-NLD pull-down and immunoprecipitation

For pull-down experiments, COS-1 cells were transfected with the pED-IRE1-NLD-His6-KDEL expression vector. Cells were harvested by washing with cold Tris-buffered saline followed by scraping into cold lysis buffer (25 mM Tris-Cl, pH 8.0, 150 mM NaCl, 0.5% Nonidet P-40, and 0.5% sodium deoxycholate). The lysate was incubated on ice for 30 min followed by centrifugation to pellet insoluble material and the lysate was incubated overnight at 4 °C with 100 µl of 10% slurry of Ni-NTA-Agarose beads (Qiagen). The beads were washed 5 times, pelleted, and re-suspended in 100 µl SDS-PAGE sample buffer, with 20 µl separated on SDS-PAGE (10% acrylamide), and followed by Immunoblot analysis using anti-COX-2, and anti-His antibodies. Experiments were done in triplicate.

For immunoprecipitation experiments, HEK293 (endogenous IRE1α) or COS-1 cells transfected with the pED-IRE1-NLD-His6-KDEL expression vector were harvested by washing with cold Tris-buffered saline followed by scraping into cold lysis buffer (25 mM Tris-Cl, pH 8.0, 150 mM NaCl, 0.5% Nonidet P-40, and 0.5% sodium deoxycholate). The lysate was incubated on ice for 30 min followed by centrifugation and 30 min incubation at 4 °C with 60 µl of 10% slurry of Protein A/G Sepharose beads (Qiagen) to preclear the lysate. The beads were centrifuged briefly to pellet and washed with five times of cold lysis buffer while the supernatant was transferred to a new Eppendorf tube. For immunoprecipitation of IRE1α, IRE1-NLD or COX-2, 2 µl of anti-IRE1α, anti-His-tag antibodies, COX-2 antibody or an IgG control were added to the supernatant and incubated with rotation overnight at 4 °C. To pull-down the antibody, 100 µl of 10% slurry of Protein A/G Sepharose beads was added and the lysate was incubated 4 hours with rotation at 4 °C. The beads were centrifuged briefly to pellet and washed five times with 1 ml cold lysis buffer. The beads were pelleted, re-suspended in 100 µl SDS-PAGE sample buffer, with 20 µl separated on SDS-PAGE (10% acrylamide), followed by Immunoblot analysis using anti-IRE1α, anti-COX-2, or anti-His-tag antibodies. Experiments were done in triplicate.

### Animal experimentations

Six-week-old male CD1 mice were purchased from Jackson and housed independently. Cyclosporine was dissolved in ethanol then diluted 1:10 in olive oil to a final concentration of 6 mg/ml. Mice (~25 g) were given a dose of 30 mg/kg/day cyclosporine by oral gavage for 21 days, with the control group given the same amount of the ethanol:olive oil mixture. The mice were euthanized 28 days after the first treatment and the kidneys were collected for analysis of XBP1 splicing by Q-PCR and COX-2 enzyme activity.

### Statistical analysis

Statistical analysis was performed using GraphPad Prism version 7.0 with a Students’ t-test used to compare the mean of two independent groups or one-way Anova used to compare the mean of three or more independent groups with a p-value determined to be significant if less than 0.05.

## Electronic supplementary material

Supplemental Figures S10-S15
