# Supplementary material for: Cyclosporine A binding to COX-2 reveals a novel signaling pathway that activates the IRE1α unfolded protein response sensor
Source: Sci Rep. 2018 Nov 12;8:16678. doi: 10.1038/s41598-018-34891-w (PMC6232179; doi:10.1038/s41598-018-34891-w)
Supplement: Supplementary file 1 — Supplemental Figures S10-S15 [file 41598_2018_34891_MOESM1_ESM.pdf]

Supplementary material to:

Cyclosporine A binding to COX-2 reveals a novel signaling pathway that activates the IRE1α unfolded protein response sensor

Jody Groenendyk, Tautvydas Paškevičius, Hery Urra, Clement Viricel, Kui Wang, Khaled Barakat, Claudio Hetz, Lukasz Kurgan, Luis B. Agellon and Marek Michalak

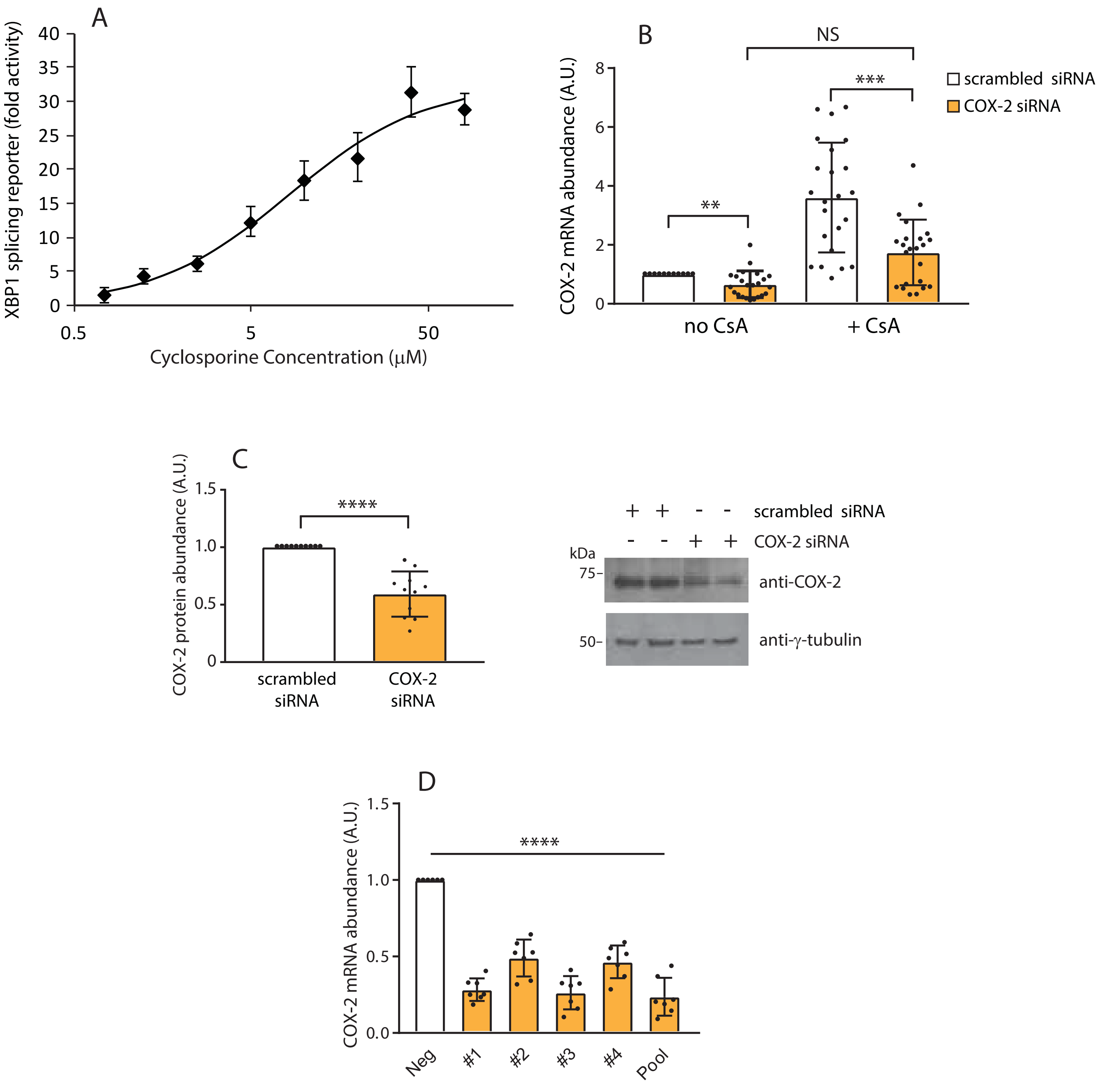

Supplemental Figure S1. Cyclosporine dose response and COX-2 mRNA silencing.
A. Cells were transfected with IRE1α splicing reporter plasmid and treated with increasing concentration of cyclosporine as indicated in the Figure. **B.** Cells were transfected with siRNA for COX-2 (*COX-2 siRNA*) or with scrambled control (*scrambled siRNA*). COX-2 mRNA abundance was normalized to GAPDH mRNA **p-value=0.0009 (n=20) ***p-value=0.0002 (n=20). NS, not significant. **C.** Immunoblot analysis of COX-2 protein in cells transfected with siRNA for COX-2 or scrambled siRNA. Protein abundance was monitored using anti-COX-2 antibodies and normalized to the loading control probed with anti-γ-tubulin antibodies ****p-value<0.0001 (n=11). The full-length gels/blots are shown in Suppl. Fig. S14.
D. Cells were transfected with single siRNA for COX-2, pool or scrambled negative control siRNA (*Neg*). Cells were harvested and mRNA for COX-2 was quantified using Q-PCR.
****p-value<0.0001 (n=7).

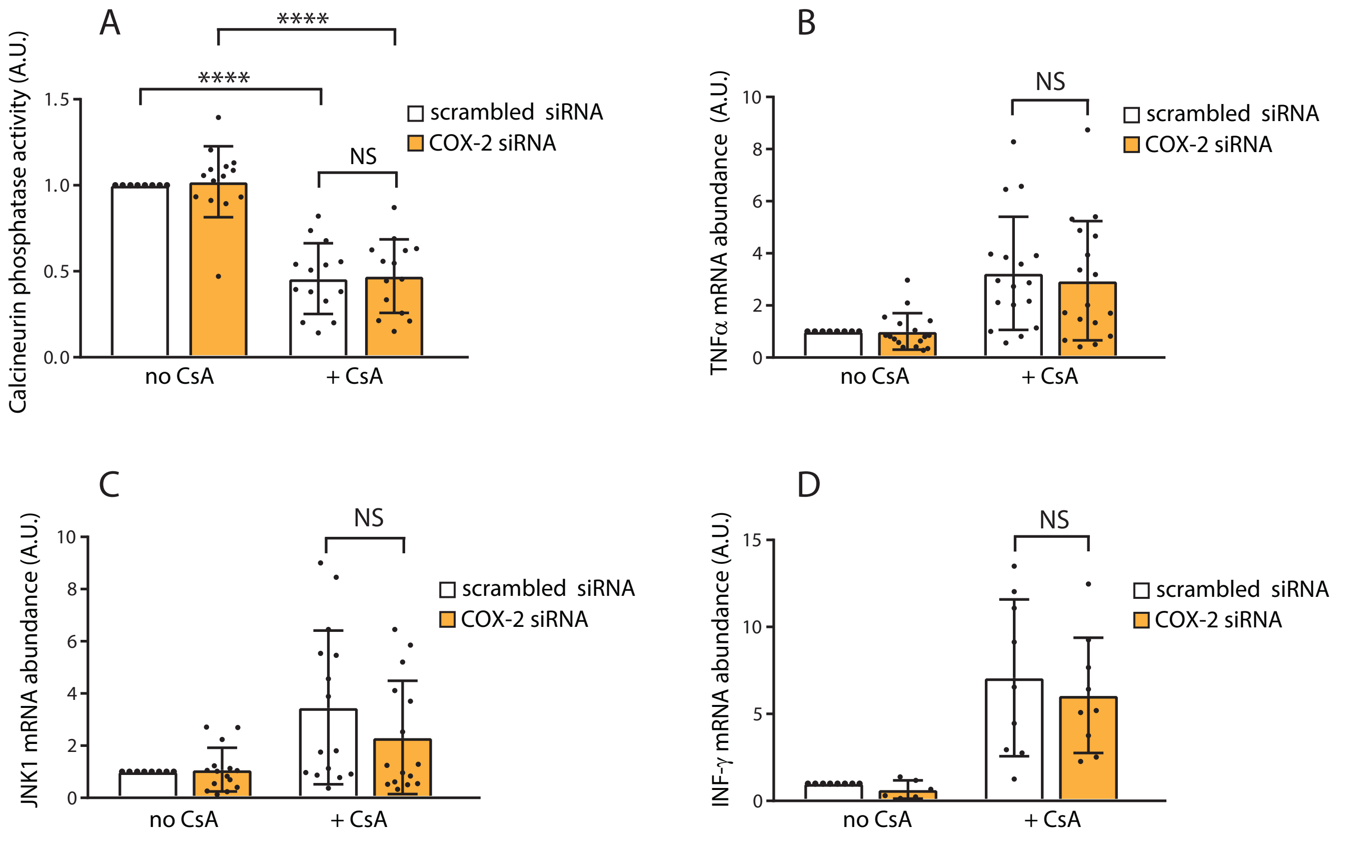

Supplemental Figure S2. Inflammation and immune responses in COX-2 silenced HEK-293 cells.
A. Cells were transfected with scrambled siRNA or siRNA for COX-2 (*COX-2 siRNA*), treated with 20 μM cyclosporine (+*CsA*) followed by a calcineurin phosphatase activity assay (n=14). ***p-value<0.0001, NS, not significant. **B.** Cells were transfected with scrambled siRNA or siRNA for COX-2 (*COX-2 siRNA*), treated with 20 μM cyclosporine (+*CsA*) followed by Q-PCR analysis of abundance of TNFα mRNA. TNFα mRNA was normalized to GAPDH mRNA (n=3). NS, not significant. **C.** Cells were transfected with scrambled siRNA and siRNA for COX-2 (*COX-2 siRNA*), treated with 20 μM cyclosporine (+*CsA*) followed by Q-PCR analysis of JNK1 mRNA. JNK1 mRNA was normalized to GAPDH mRNA (n=3). NS, not significant. **D.** Cells were transfected with scrambled siRNA and siRNA for COX-2 (*COX-2 siRNA*), treated with 20 μM cyclosporine (+*CsA*) followed by Q-PCR analysis of INF-α mRNA. INF-γ mRNA was normalized to GAPDH mRNA (n=3). NS, not significant.

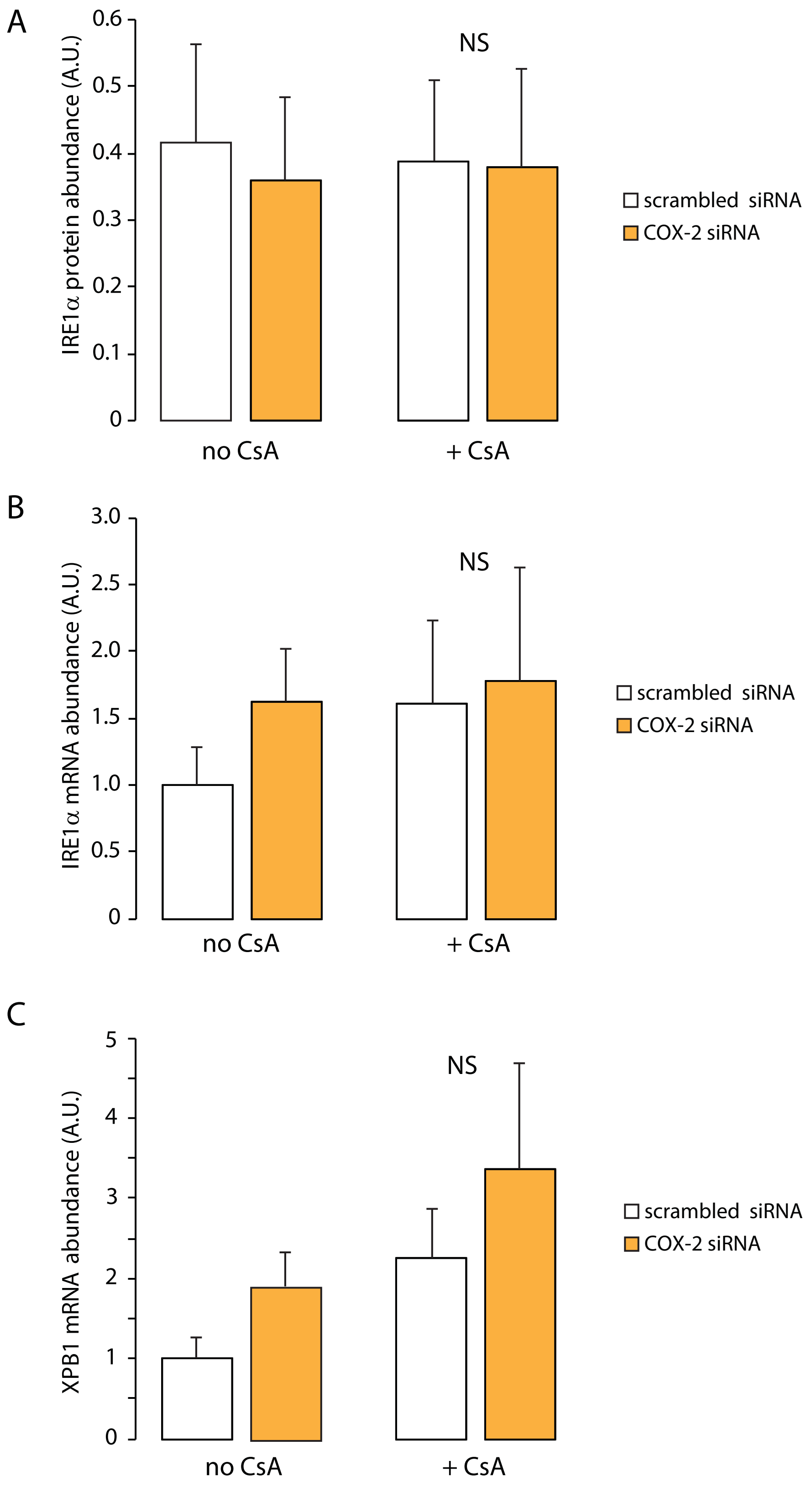

Supplemental Figure S3. Abundance of IRE1α and XBP1 in cyclosporine treated cells.
Cells were transfected with scrambled siRNA or siRNA for COX-2 (*COX-2 siRNA*), treated with 20 μM cyclosporine (+*CsA*) followed by immunoblot analysis of IRE1α protein (**A**) and Q-PCR analysis of IRE1α (**B**) and XBP1 (**C**) mRNA (n=3). NS,not significant.

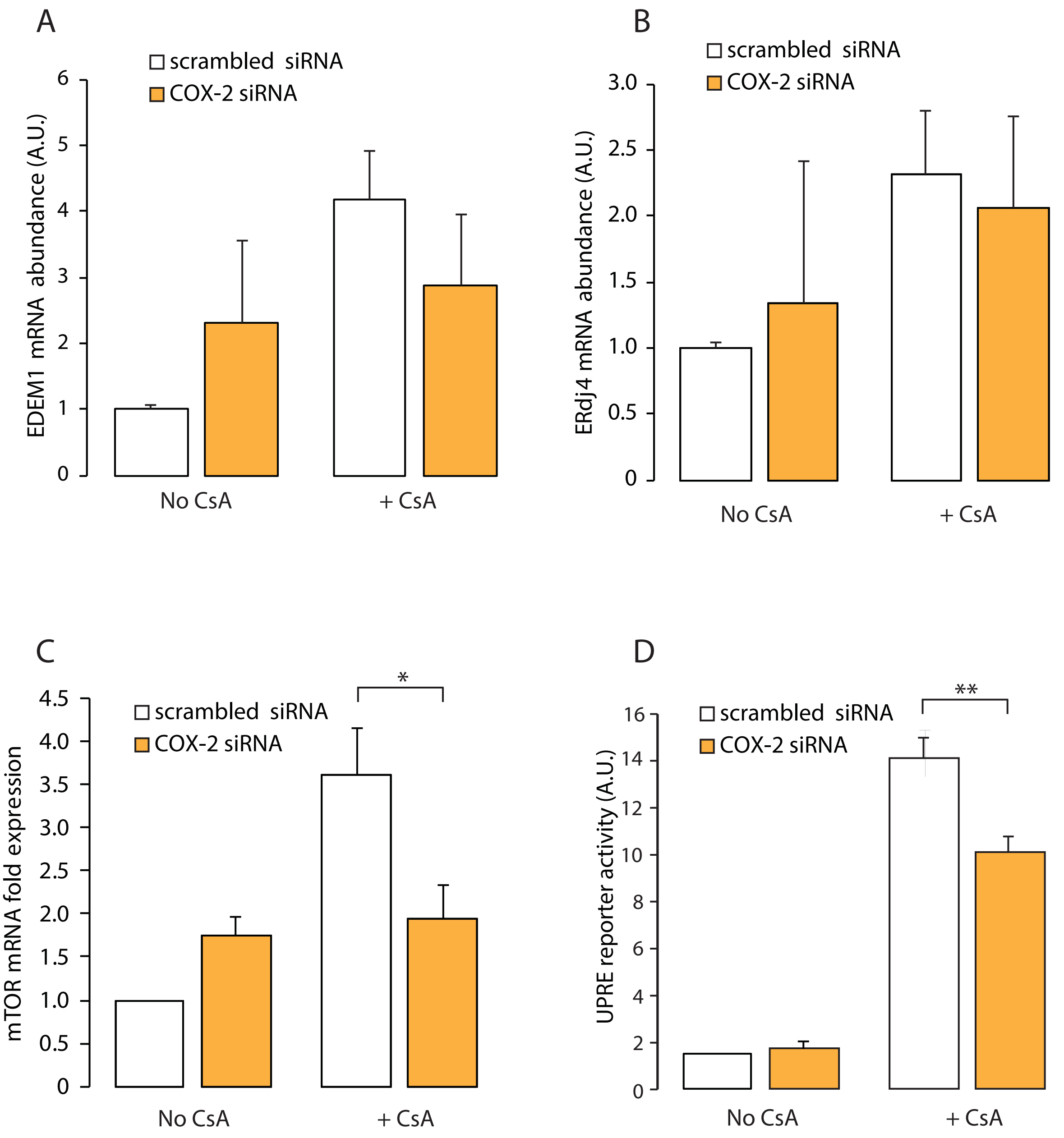

Supplemental Figure S4. Analysis of XBPs target genes.
Cells were transfected with scrambled siRNA or siRNA for COX-2 (*COX-2 siRNA*), treated with 20 µM cyclosporine (+*CsA*) followed by Q-PCR analysis. **A.** EDEM1 mRNA (n=3). **B.** ERdj4 mRNA abundance (n=3). **C.** mTOR mRNA abundance, *p*-value=0.0471 (n=3). **D.** UPRE reporter activity in cells transfected with scrambled siRNA and siRNA for COX-2 (*COX-2 siRNA*) and treated with 20 µM cyclosporine (+*CsA*) (n=3). Data was normalized to GAPDH mRNA (n=3).

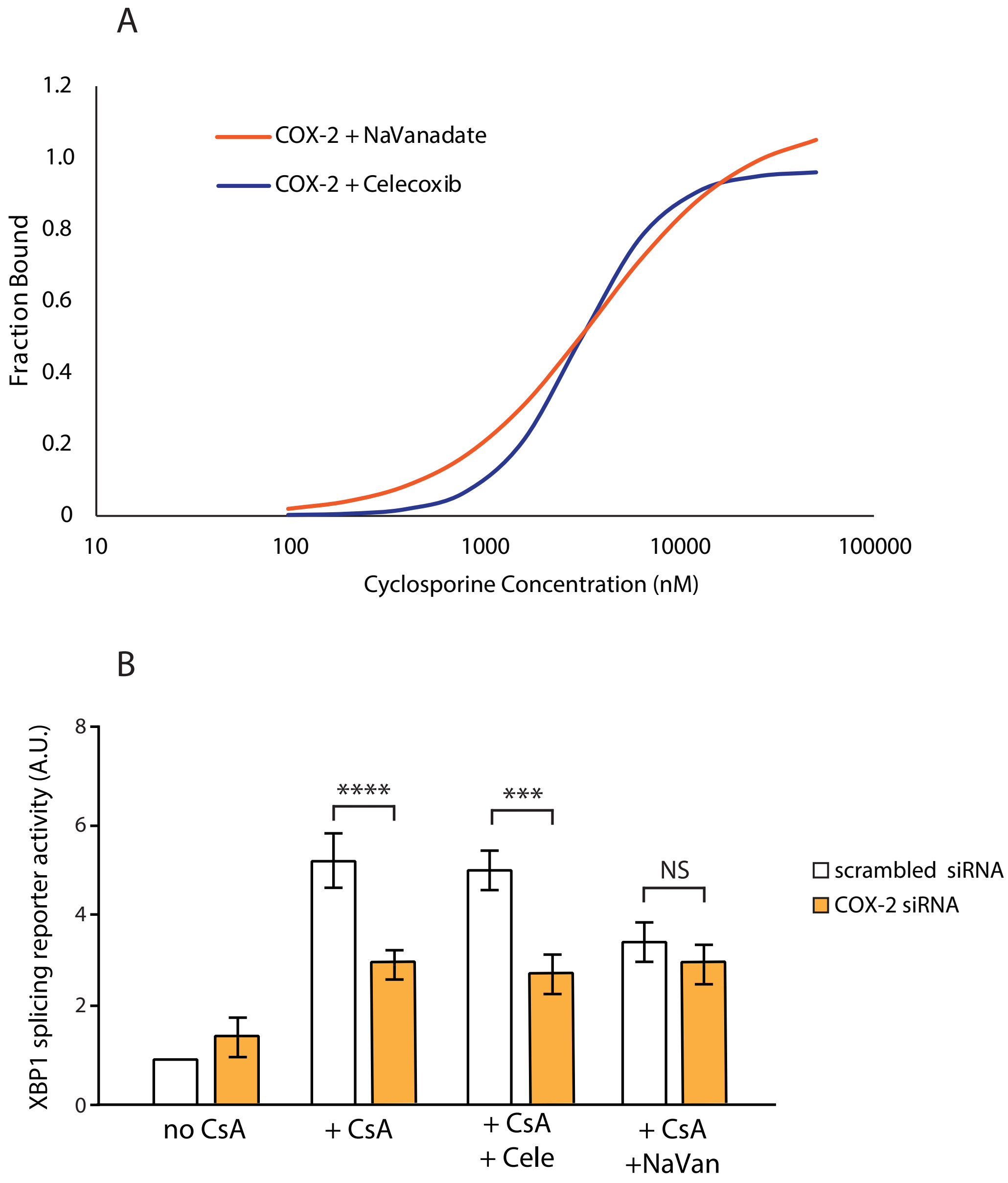

Supplemental Figure S5. Effects of COX-2 inhbitors on XBP1 splicing.
A. Binding of cyclosporine to COX-2 in the presence of sodium vanadate (*COX-2 + NaVanadate*) or celecoxib (*COX-2 + Celecoxib*). **B.** Cells were transfected with scrambled siRNA or siRNA for COX-2 (*COX-2 siRNA*), treated with 20 μM cyclosporine (+*CsA*) or 20 μM cyclosporine + celecoxib (+*CsA*+*Cele*) or 20 μM cyclosporine + sodium vanadate (+*CsA*+*NaVan*). ****p-*value*<0.0001 (n=20); ***p-*value*<0.0001 (n=3).

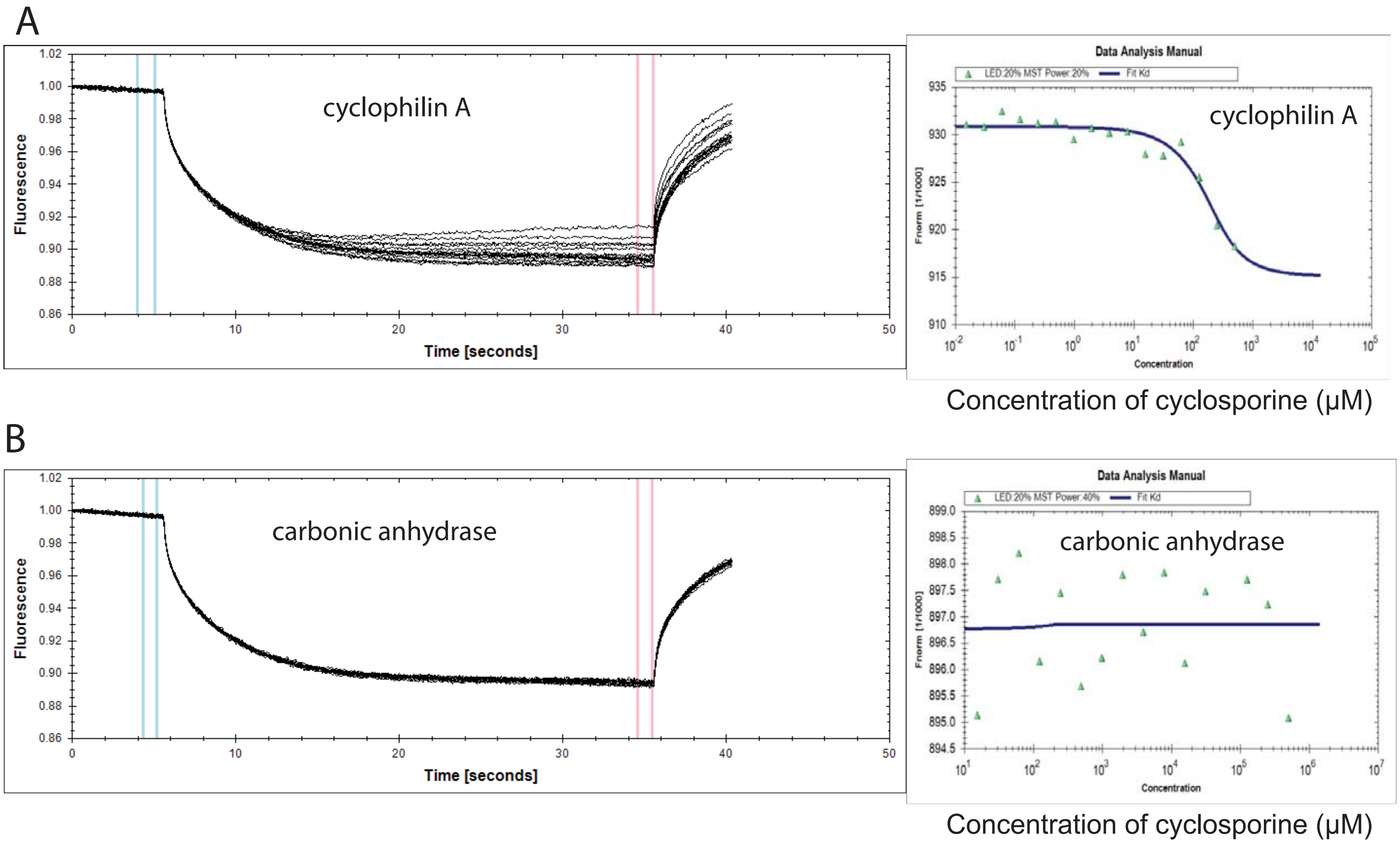

Supplemental Figure S6. Positive and negative controls for cyclosporine binding using MST. MST analysis of cyclosporine binding to cyclophilin A (positive control) (**A**) or carbonic anhydrase (negative control) (**B**) (n=3).

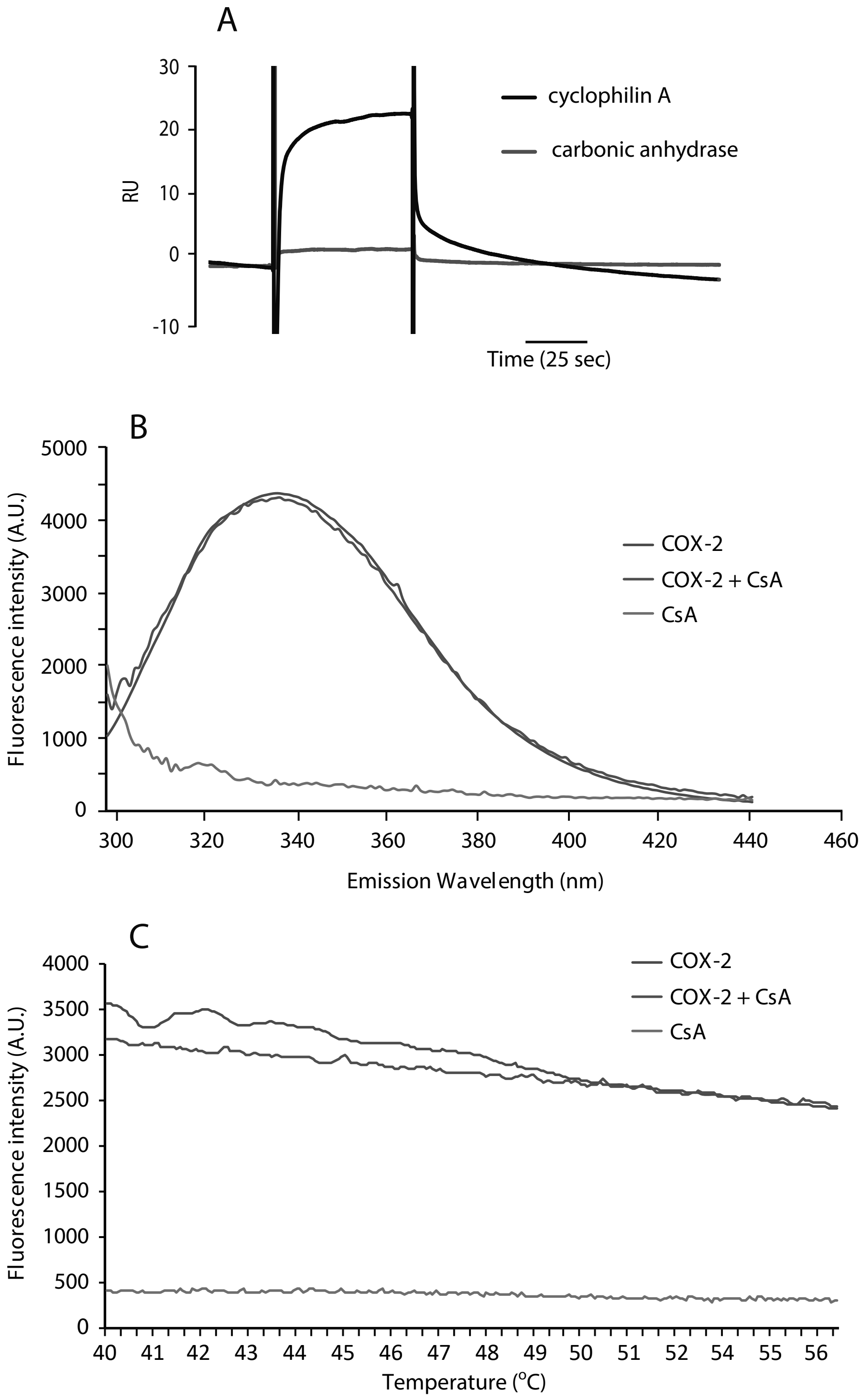

Supplemental Figure S7. Controls for cyclosporine binding, COX-2 intrinsic fluorescence and thermal stability. **A.** Cyclophilin A or carbonic anhydrase was covalently linked to a CM5 chip and 20 µM cyclosporine was flowed over the chip to monitor positive binding (*cyclophilin A*) or negative binding (*carbonic anhydrase*) (n=3). **B.** Intrinsic fluorescence of COX-2 in the absence and presence of 20 µM cyclosporine (+*CsA*) (n=3). Ex. at 286 nm. **C.** Thermal stability analysis of COX-2 in the absence and 20 µM cyclosporine (+*CsA*) (n=3). Ex. at 286 nm; Em. at 340 nm.

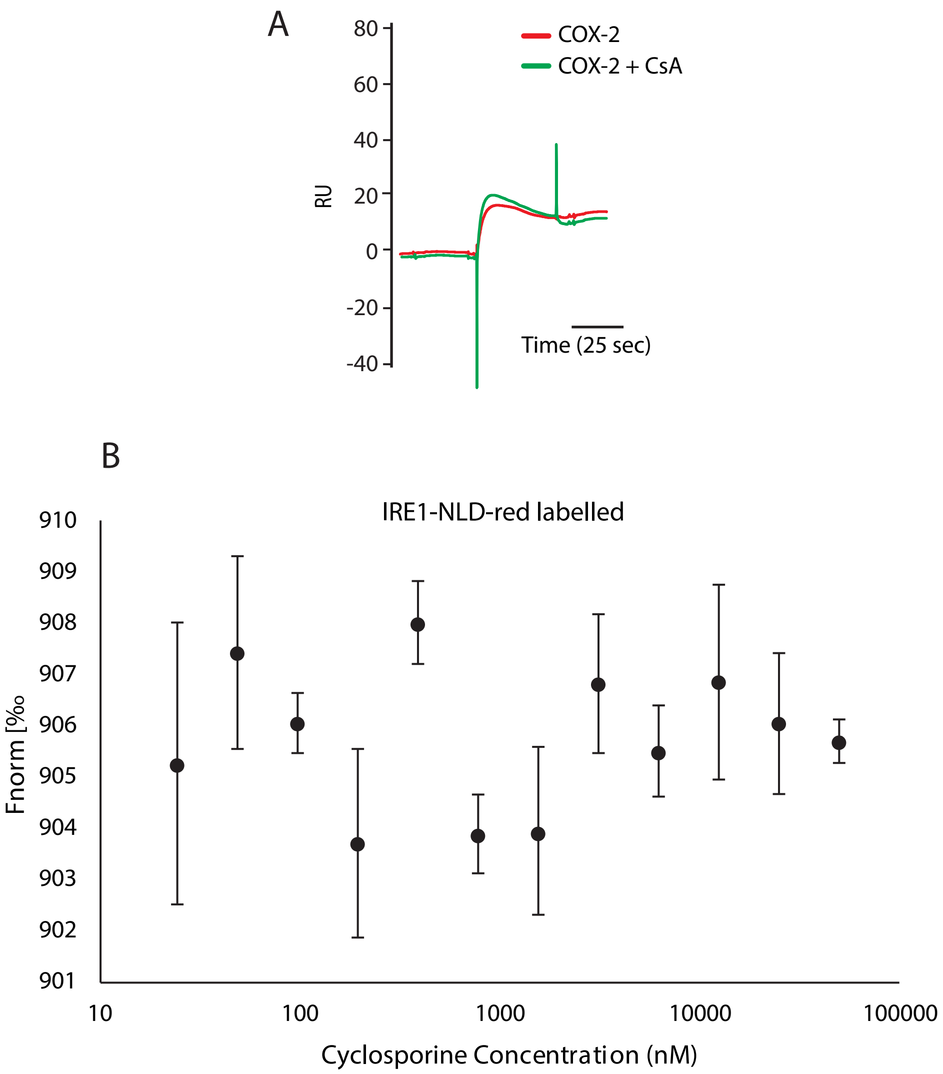

Supplemental Figure S8. COX-2 and cyclosporine binding to IRE1-NLD.
A. IRE1-NLD was linked to a CM5 chip followed by analysis of COX-2 binding in absence or presence of 20 µM cyclosporine (+CsA) (n=3). **B.** MST analysis of cyclosporine bining to IRE1-NLD.

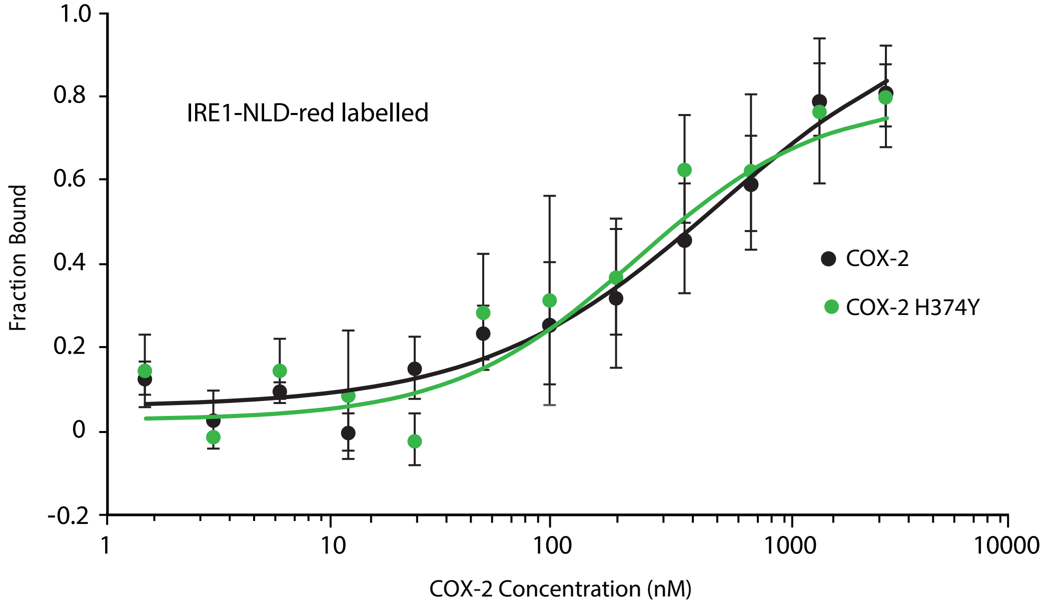

Supplemental Figure S9. Analysis of COX-2 H374Y mutant. Purified IRE1-NLD protein was covalently labeled with a red fluorescent tag and incubated with increasing amounts of purified COX-2 or COX-2 H374Y mutant protein followed by Microscale Thermophoresis (MST).

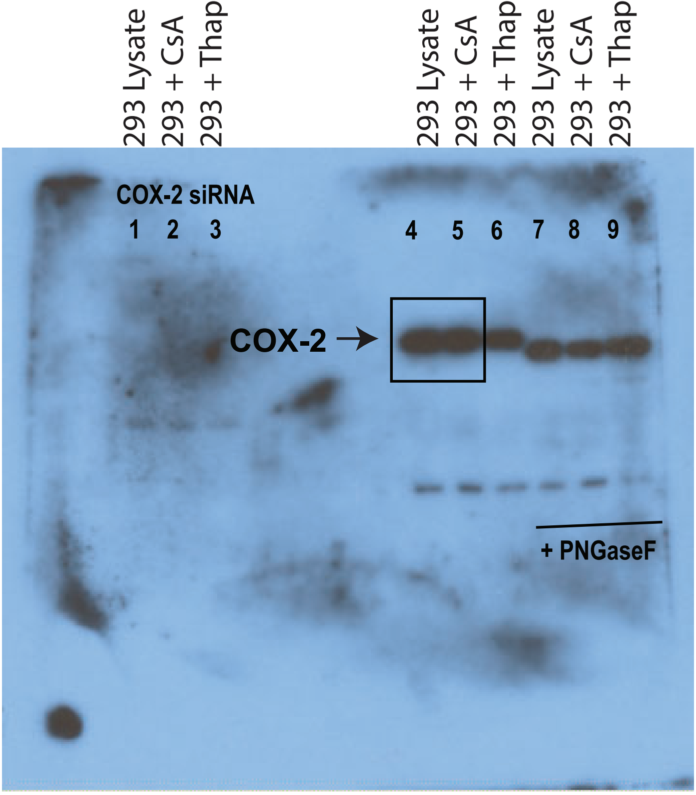

anti-COX-2

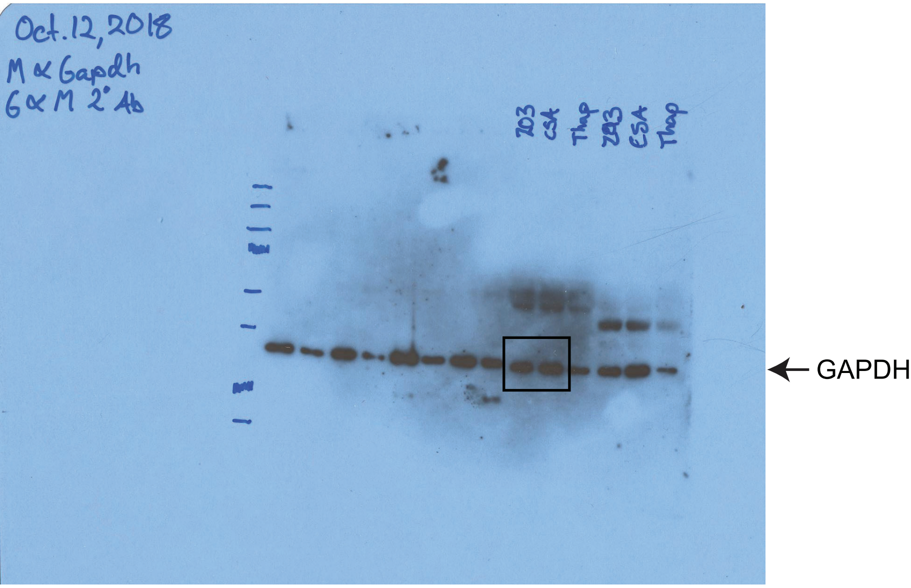

anti-GAPDH

Figure S10. The full-length blots for Figure 1E

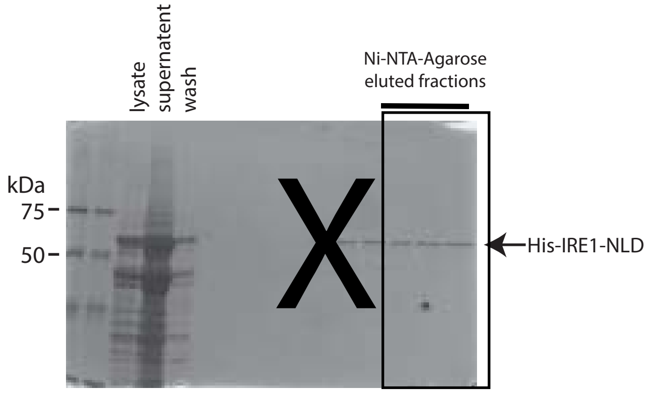

Figure S11. The full-length blots for Figure 4A

Figure 5A TOP

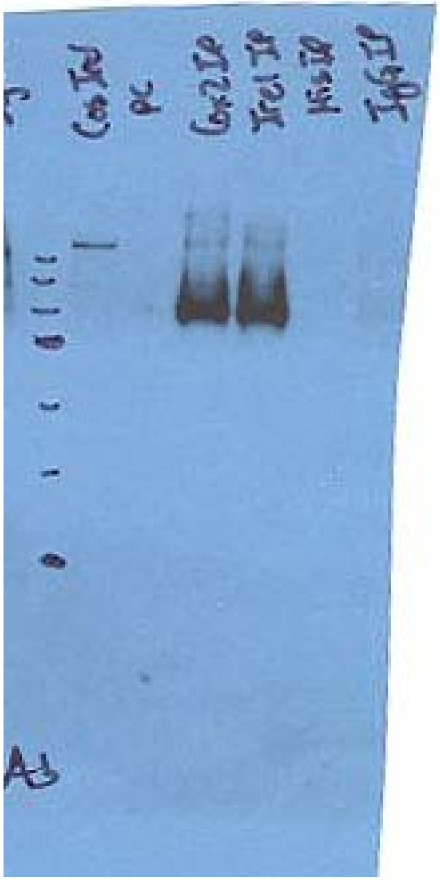

Anti-IRE1

Figure 5A BOTTOM

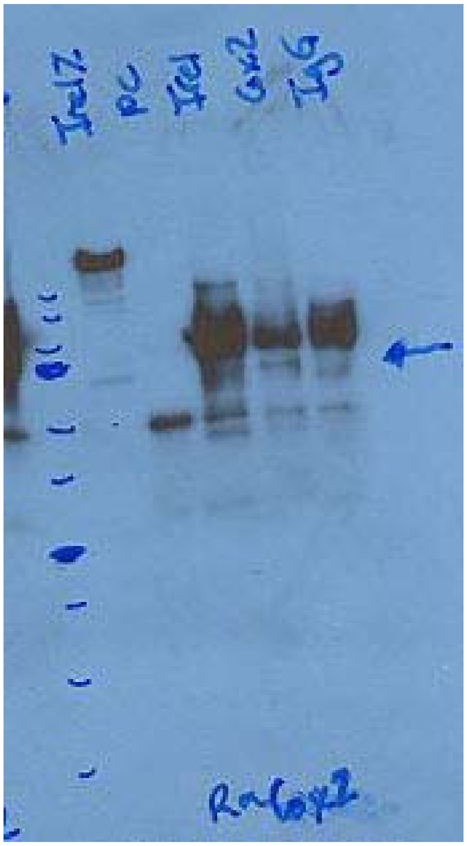

Anti-COX_2

Figure 5B

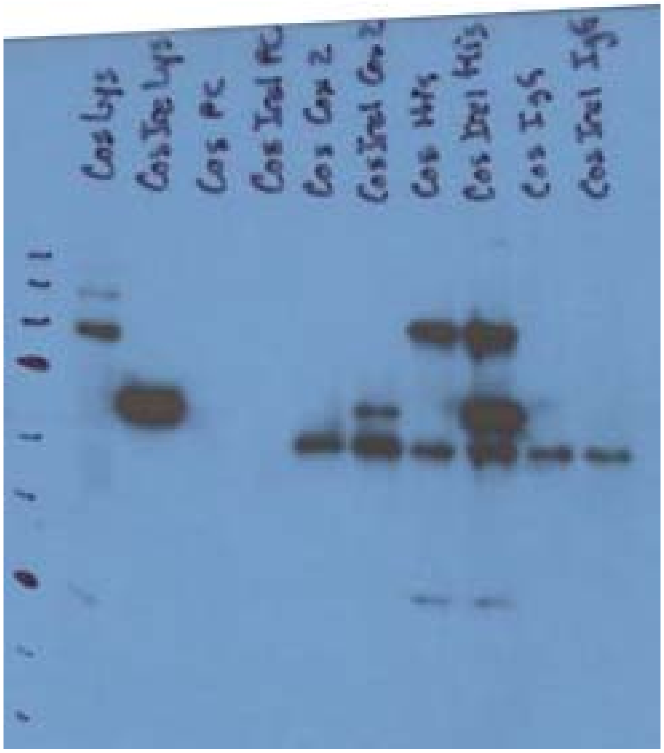

Anti-His

Figure S12. The full-length blots for Figure 5A and 5B

Figure 5C TOP

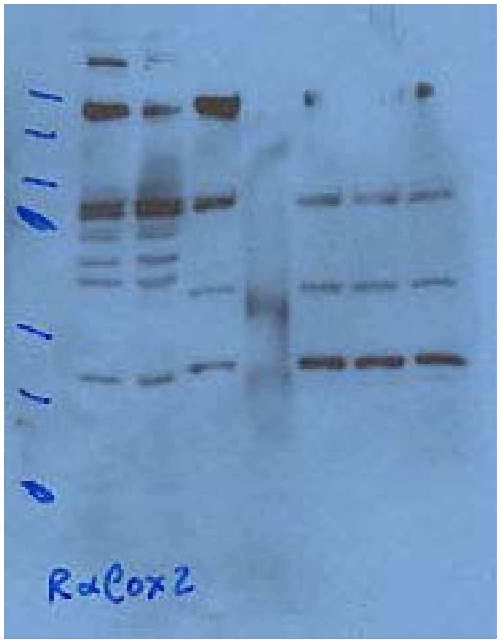

Anti-COX-2

Figure 5C BOTTOM

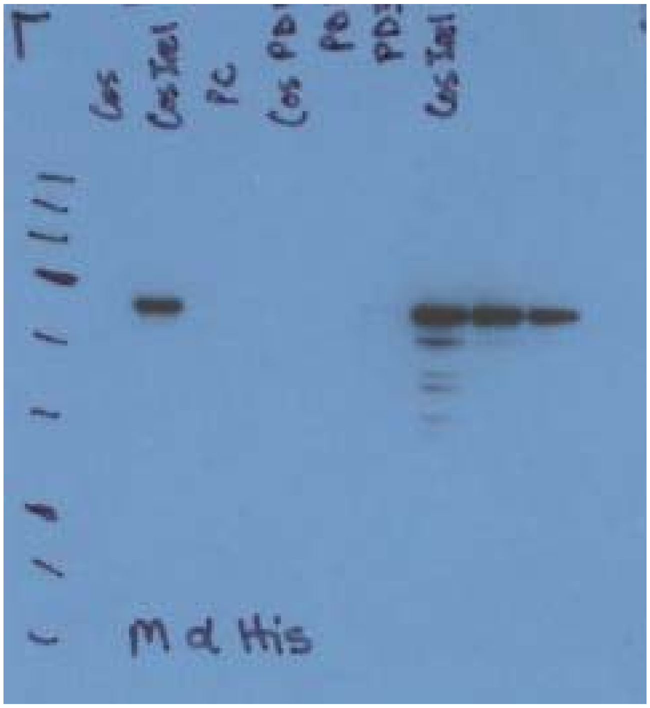

Anti-His

Figure S13. The full-length blots for Figure 5C

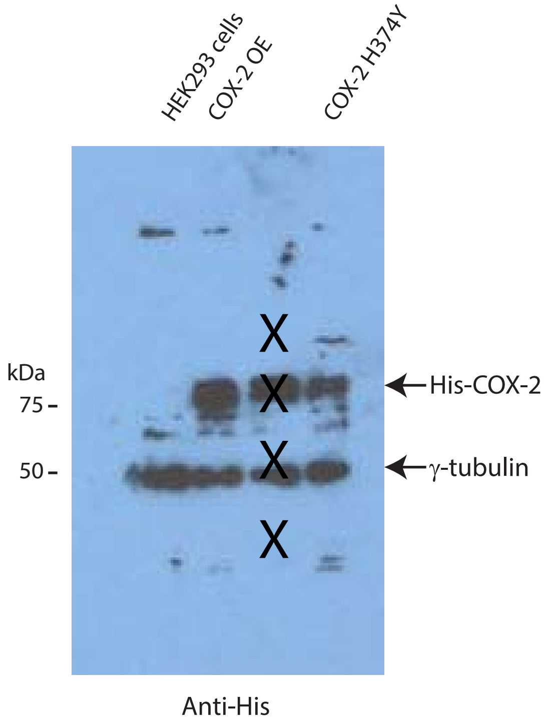

Figure S14. The full-length blots for Figure 6A

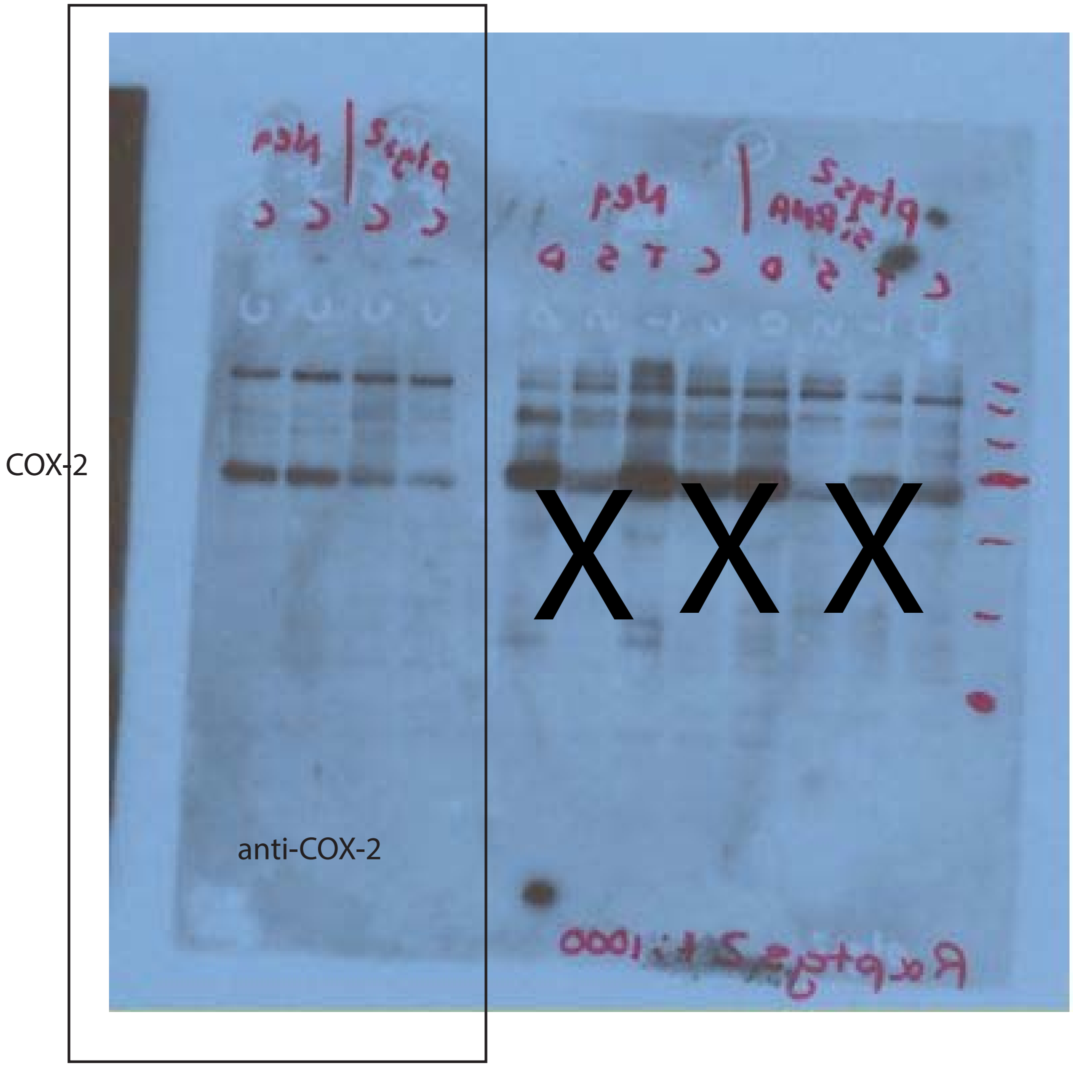

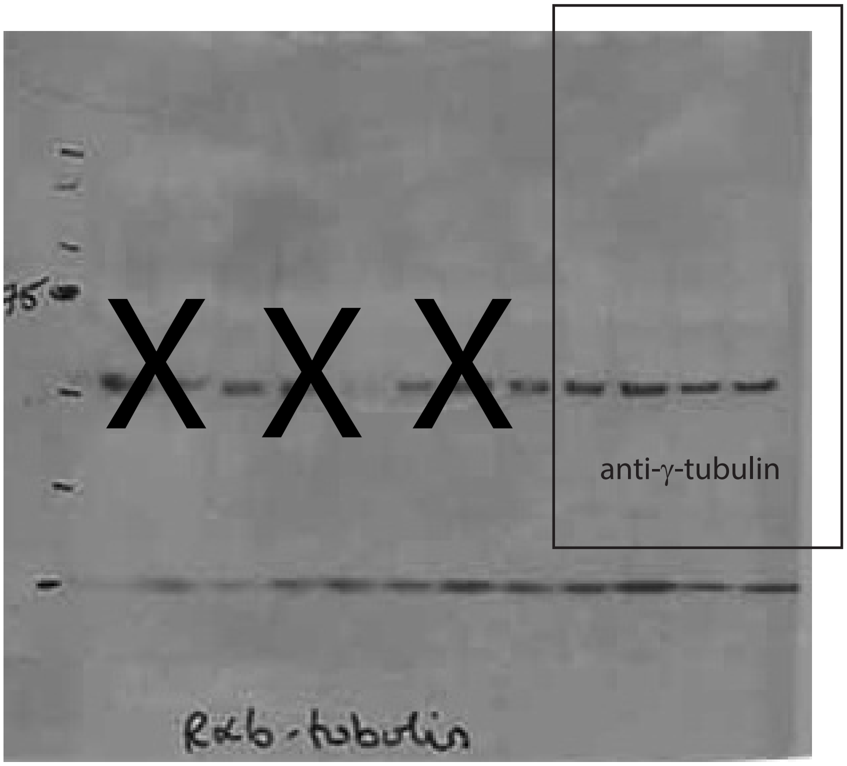

Figure S15. The full-length blots for Figure S1C
